# How does interprofessional education affect attitudes towards interprofessional collaboration? A rapid realist synthesis

**DOI:** 10.1007/s10459-024-10368-6

**Published:** 2024-09-23

**Authors:** Jean Anthony Grand-Guillaume-Perrenoud, Eva Cignacco, Maura MacPhee, Tania Carron, Isabelle Peytremann-Bridevaux

**Affiliations:** 1 Department of Health Professions, Bern University of Applied Sciences, Bern, Switzerland; 2 School of Nursing, University of British Columbia, Vancouver, Canada; 3 Unisanté, University Center for Primary Care and Public Health, Department of Epidemiology and Health Systems, University of Lausanne, Lausanne, Switzerland

**Keywords:** Interprofessional education, Interprofessional collaboration, Healthcare professionals, Attitudes, Attitude development, Realist synthesis

## Abstract

Interprofessional collaboration (IPC) in healthcare is regarded as important by professionals, as it increases the quality of care while decreasing costs. Interprofessional education (IPE) is a prerequisite for IPC and influences learners’ attitudes, knowledge, and collaboration skills. Since attitudes shape behavior, understanding how they are formed is crucial for influencing IPC in learners’ professional practice. We investigated what kind of IPE works, for which students, how, and in what circumstances to develop positive attitudes towards IPC. Using realist synthesis, we extracted causal mechanisms that produce positive attitude outcomes and the conducive contexts that trigger them. Our analysis resulted in six plausible context-mechanism-outcome configurations that explain positive attitude development. Positive IPC attitudes are more likely to arise in contexts where IPE provides time and facilities for formal and informal interactions, as this allows learners to get to know each other both professionally and personally, fostering trust, respect, and mutual liking. Additionally, positive attitudes are more likely in contexts where the IPE curriculum is perceived as career-relevant and boosts confidence. Key mechanisms of positive attitude development include getting to know the other learners professionally and personally, experiencing positive affect during IPE, and learners experiencing mutual dependence. Sustained positive attitudes are more likely to develop when there is organizational support for IPC and professionals attend IPE on an ongoing basis, allowing the attitudes and values expected in IPC to be positively reinforced and eventually integrated into the learners’ personal value system.

**Supplementary Information:**

The online version contains supplementary material available at 10.1007/s10459-024-10368-6.

## Background

Interprofessional collaboration (IPC) in healthcare is regarded as important by healthcare professionals and increases quality of care while decreasing costs (Schmitz et al., 2017; Wei et al., 2020, 2022). It involves two or more health or social care professions providing care and can positively impact several domains, including healthcare access for users, patient satisfaction, and length of hospital stay (Reeves et al., 2017; Wei et al., 2022). For providers, IPC can contribute to facilitating information flow, fostering professional exchange between professions and increasing workplace satisfaction (Reeves et al., 2017; Wei et al., 2022). IPC is often defined as the coordinated effort of various healthcare professionals to deliver comprehensive, quality care to patients and communities across multiple settings (WHO, 2010). The IPC construct is conceptually proposed to comprise four interrelated dimensions: Interprofessional education (IPE), organizational requirements, practical interprofessional collaboration, and the effects of such collaboration (Wagner et al., 2019). IPE is commonly described as consisting of situations that allow students or professionals to “learn with, from and about each other to improve collaboration and the quality of care” (CAIPE, 2017, p. 14).

With IPE as a prerequisite of IPC (Spaulding et al., 2021; Wagner et al., 2019), educational institutions training healthcare professionals have a unique opportunity to cultivate IPC in their future workforce. IPE has been found to influence learners’ attitudes, knowledge, and skills of collaboration (Campion-Smith et al., 2011; Makowsky et al., 2009; Reeves et al., 2013; Sargeant et al., 2011), which are plausible vectors through which IPE affects IPC. Learning is often defined in terms of long-term behavior changes that are preceded by changes in learner attitudes (De Houwer et al., 2013). This points to attitudes as a feasible target for educational efforts and is supported by theoretical propositions in Ajzen’s (1991) Theory of Planned Behavior. As a form of academic learning, IPE may favorably affect learners’ attitudes toward members of other professional groups and attitudes towards IPC. Indeed, IPE explicitly aims to improve learners’ knowledge, skills, and attitudes (Stephens & Ormandy, 2018).

Despite the theory-based expectation that IPE affects attitudes towards IPC, the contextual factors and underlying mechanisms by which IPE affects attitudes are not always clearly delineated in the literature. Much of the empirical research focuses on predictor variables that are statistically associated with interprofessional attitude score improvements after conducting IPE (e.g., Biehle et al., 2019; Bloomfield et al., 2021; Fusco & Foltz-Ramos, 2018; Lockeman et al., 2017). However, literature syntheses show mixed results of IPE’s impact on attitudes (Berger-Estilita et al., 2020a, 2020b; Spaulding et al., 2021). The contradictions are partially attributed to the variety of different IPE initiatives and the lack of sufficiently sophisticated evaluation methodology (Thistlethwaite, 2012). There also appear to be only a few reviews summarizing the evidence of IPE’s impact on attitudes applying a conceptual model (e.g., Hammick et al., 2007; Reeves et al., 2016; Spaulding et al., 2021). These shortcomings highlight the need for realist approaches (Wong et al., 2012), which consider the contextual factors in which attitude change occurs, as well as the mechanisms associated with positive attitude change. By developing a more fine-grained understanding of how IPE works to develop positive attitudes, we can support the development of more targeted IPE curricula and more conducive learning environments.

As a theory-driven method, realist synthesis enriches data analysis with existing theory (Jagosh, 2019). At least four branches of IPE literature can be discerned which address theory, theory development, or theory application. The first branch consists of competency frameworks, such as of the Interprofessional Education Collaborative (IPEC, 2016) and the Canadian Interprofessional Health Collaborative (CIHC, 2010), which describe some of the theoretical assumptions of how IPE works. The second branch of literature consists of empirical studies which are framed or interpreted from a specific theoretical perspective, for instance the Theory of Planned Behavior (e.g., Keshmiri et al., 2020; Przymuszala et al., 2023) or Social Learning Theory (e.g., Chen et al., 2022; Wu et al., 2022). These middle-range theories (MRTs) are frameworks that organize hypotheses and facilitate the development of empirically testable propositions (Boudon, 1991; Merton, 1949). A third literature branch evaluates the contribution of theory to IPE curriculum design, delivery, and evaluation (e.g., Anderson et al., 2016; Hean et al., 2018). A fourth branch consists of literature reviews which contribute to theory development by summarizing the effects of IPE on learning and other outcomes (e.g., Reeves et al., 2017; Spaulding et al., 2021).

The application of these various forms of theory can guide analysis and fill evidence gaps concerning mechanisms with theoretical assumptions (Jagosh, 2019). Drawing on various sources of data, the realist heuristic of context-mechanism-outcome configurations (CMOCs) can be applied to uncover hidden causal mechanisms producing positive attitude outcomes and the conducive contexts that trigger them (Jagosh, 2019, 2020; Pawson & Tilley, 2004).

Even though realist synthesis provides a useful heuristic for theory development (Jagosh, 2019), theory inevitably remains undertermined by evidence (Turnbull, 2018). That is, often multiple theories are equally well suited to explain a specific set of data, a fact that has also been echoed in the realist synthesis literature (Wong et al., 2010). This circumstance requires one to consider a range of criteria when developing IPE theory or applying an existing one to explain IPE phenomena. These criteria may also serve as additional heuristics to guide theory development. For instance, one might consider the context of theory application, i.e., whether the theory is to serve in IPE curriculum design, delivery, or evaluation (Hean et al., 2018). Hean et al. (2016) have also proposed considering aspects of theoretical quality such as parsimony, testability, operational adequacy, empirical adequacy, and quality of theory application. Theory selection may also depend on dimensions such as which group of stakeholders a theory is intended to serve (e.g., curriculum developers, facilitators), when in the IPE intervention a theory might be applicable (i.e., pre- or post-registration), the learning environment, the utility of a theory in helping achieve an intervention goal (Hean et al., 2012), or its level of analysis (i.e., individual, group, or system level) (Hean et al., 2018). Ultimately, theory provides IPE curriculum developers with the theoretical foundations to support the IPE activities being implemented (Hean et al., 2018) and to help reflect on existing practices (Hean et al., 2012). Thus, it may be useful to take an instrumental approach (Suppes, 2014) or tool box approach (Hean et al., 2012) to selecting a theory, in order to pick one that is best suited to describe, explain, or predict phenomena of interest, make the links explicit between an intervention and an outcome (Hean et al., 2016), and inform the social processes underlying IPE curricula (Hean et al., 2018). It is also necessary to ensure that developed IPE theory is useful for practioners, for instance in curriculum development, IPE delivery, or evaluation, and the links between theory and practice clearly put forward (Hean et al., 2016).

Of the criteria described, we focus our theory development and MRT selection on their ability to describe and explain positive attitude development, ensuring explicit links to IPE, and providing useful recommendations for IPE practitioners. The overarching research questions we investigated were: How does IPE work to positively influence student attitudes toward IPC, IPE and professionals from other healthcare fields? For which learners is IPE effective? Under what circumstances does IPE work?

## Methods

We conducted a rapid realist synthesis to identify CMOCs that link IPE to learner attitudes towards IPE, IPC, and professionals from other disciplines. As a method that provides the tools to investigate “what works, for whom, how, and in what circumstances” (Pawson et al., 2005, p. 32), realist synthesis is particularly suited to addressing the IPE literature’s shortcoming by using the CMOC heuristic to uncover the hidden mechanisms (Jagosh, 2019) through which IPE affects learner attitudes. Applying this form of theorizing called retroduction, we began with the outcomes and analytically worked our way backward to the conditions required to produce them (Jagosh, 2020, p. 129). With changes in attitudes as our outcome, we looked for potential mechanisms that could be generating changes in attitudes. We also paid attention to intermediate outcomes that might be linked to attitude change. We were mindful that outcomes cannot always be predicted by deterministic theory. Instead, contexts can be expected to shape decisions and behavior in ways that are partially predictable because people are likely to make similar decisions or behave similarly given a certain set of circumstances (Wong et al., 2010). Thus, we analyzed the data looking for descriptions of contexts, as a context provides the trigger for every mechanism (Dalkin et al., 2015; Greenhalgh & Manzano, 2022) that leads to changes in attitudes. We used multiple sources of evidence, as realist synthesis does not privilege specific sources of evidence over others (Duddy & Roberts, 2022; Wong, 2018), combining information gained from qualitative interviews with IPE subject matter experts (SMEs) and published studies. Finally, we looked for MRTs to support our proposed mechanisms and used them to explain our findings, building on their theoretical propositions for how and why specific mechanisms are necessary to yield intended outcomes. The Theory of Planned Behavior (Ajzen, 1991) is one example of an MRT we used.

Endeavoring to complete the review within approximately one year, we set fixed dates to complete the various project phases. This limited the length of time available for analysis iterations, group discussions, and developing versions of CMOCs and program theories. This paper, a synthesis of literature and SME input, follows the RAMESES publication standards for realist reviews (Wong et al., 2013).

## Data collection

Data collection comprised three elements: 1) a preliminary literature search, 2) qualitative interviews with subject matter experts, and 3) a realist synthesis of documents contributing to development and refinement of a PT and CMOCs.

### Preliminary literature review

The preliminary literature review targeted existing systematic reviews on the effects of IPE on attitudes, as this body of literature provides a comprehensive overview of the empirical literature and and may point to potential mechanisms by which IPE affects attitudes. This literature supported initial program theory (IPT) development of how IPE works to affect students’ attitudes towards IPE, IPC, and professionals outside of one’s own profession. This preliminary review was also used to develop an interview guide for subject matter experts (SMEs) and search terms for the realist review.

### Qualitative interviews

The interview guide included questions about the components of IPE to better understand the contextual factors (e.g., teaching environment and conditions, student background and characteristics) and to explore potential underlying mechanisms associated with attitude change in students. SME interviews were conducted in Swiss German with healthcare professionals in Switzerland who have backgrounds in nursing (2 SMEs), medicine (1 SME), physiotherapy and neuropsychology (1 SME). While three SMEs are involved in interprofessional curriculum development and teaching, one SME heads an interprofessional clinic and serves on the board of directors of a large university hospital. All persons interviewed are mid- to late-career professionals with extensive experience in interprofessional practice. Three SMEs were actively involved in interprofessional teaching at the time of the interview. The interviews took place between November 2021 and July 2022. Two interviews took place online using the MS Teams video platform, and two took place in the respective offices of the SMEs.

### Realist synthesis

Based on the preliminary literature review and SME interviews, a realist synthesis search strategy was developed. A Population-Intervention-Comparison-Outcome (PICO) framework was used to organize eligibility criteria (Table 1) for a systematic database search of MEDLINE (PubMed), CINAHL, PsycINFO, and Social Science Citation Index. The target population included pre- and post-registration learners so as to provide insights into IPE impacts on attitudes in the formative stages of professional development, as well as insights into IPE’s potential impact on attitude maintenance after entry into practice. IPE and multiprofessional education were included as interventions to be studied, under the theoretical assumption that they may share some similar mechanisms and some unique mechanisms that contribute to positive attitudes. The broader selection criteria chosen may allow studies to be found which permit a comparative analysis of group outcomes, a broader generalization of findings, and the potential discovery of IPE mechanisms of effect common to both contexts. The search strategy was developed with the support of a medical librarian at a medical university library. Four groups of search terms were combined: terms related to 1) education, 2) attitude, 3) practice, and 4) impact (Table 2). The full search strategy is included in Supplementary File 1. Various types of publications and all study designs (quantitative, qualitative, mixed methods, review) were included if published between January 1, 2010 and June 13, 2022. Publications were assessed based on the realist synthesis criteria of relevance and rigor (Dada et al., 2023; Wong et al., 2013). Relevance refers to how well the study provides answers to the research question. Rigor refers to the trustworthiness of the research method used to arrive at the study conclusions. To perform these assessments, authors individually reviewed assigned papers for content that could contribute to the research question and for potential methodical issues that could cast doubt on a study’s findings. Critical appraisal tools were not applied, as we prioritized the inclusion of substantive content.

**Table 1:** Eligibility criteria

PICO	Inclusion Criteria	Exclusion Criteria
Population	Studies with students of medicine or an allied health profession (e.g., dietetics, nursing, midwifery, physiotherapy, psychology) Studies with medical or allied health professionals	Studies with social care students or professionals Studies with students or professionals who do not work in healthcare
Intervention	IPE course, i.e., a course specifically intended to improve IPC by allowing students/professionals to learn from, with and about each other Multi-professional education, i.e., joint learning alongside another profession	Non-education-related interventions, e.g., team building exercises Interventions not targeting students or members of at least two professions
Comparison	Studies with students or professionals from medicine or allied health who have attended courses with members of other professions, but which are not IPE courses Studies with students or professionals from medicine or allied health who have not attended any courses with students or members of other professions	Studies that make a comparison with students or professionals outside of the medical or healthcare professions
Outcomes	Attitudes of students or professionals towards IPE, IPC, and attitudes towards students or professionals outside of their own field	General work, workplace, or work-related attitudes General attitudes towards education Achievement motivation

**Table 2:** Configuration of search terms

*Terms relating to education:* (Interprofessional education (MeSH) OR Interprofessional training OR Multi-professional education OR Multi-professional training) AND
*Terms relating to attitude:* (Attitude of health personnel (MeSH) OR Attitudes OR Interpersonal relations (MeSH) OR Perceptions OR Values) AND
*Terms relating to practice:* (Interprofessional collaboration OR Interprofessional practice OR Interprofessional teams) AND
*Terms relating to impact:* (Impact OR Effect OR Association OR Link)

**Fig. 1:**
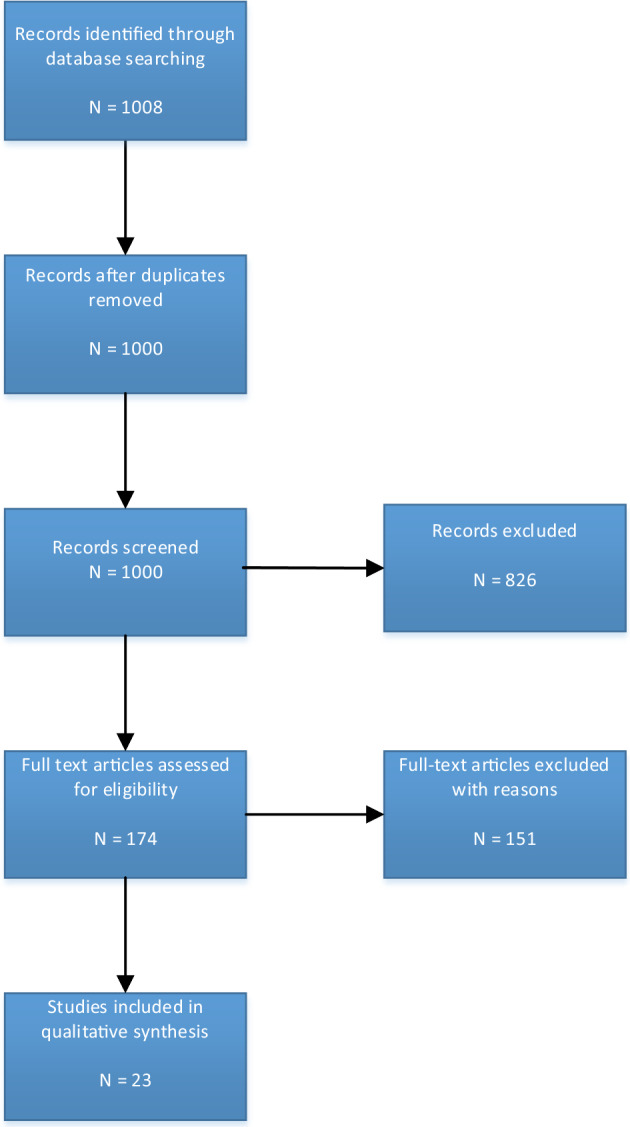
Study selection process

The study selection process is illustrated in Fig. 1.

### Data extraction and organization

A data extraction file in the form of an Excel table was created to document study characteristics (e.g., study sample, setting, study objectives, putative contexts, mechanisms, and outcomes) (Table 4). Another table was used to collect and annotate verbatim text segments from studies that appeared to contain contexts, mechanisms, or outcomes and potential linkages between them, for instance a context-mechanism, a mechanism-outcome, a context-outcome, or a full context-mechanism-outcome linkage. MRTs explicitly cited in the studies were noted separately for later reference. The verbatim text segments were thematically coded, which formed our conceptual “buckets”, for instance as “IPE characteristics” or “IPE learner development/transformation” (Supplementary File 2). The conceptual buckets served as analytic containers for grouping text segments that appeared to discuss the same phenomenon. After all studies and all their informative text segments had been thematically coded, they were sorted according to the conceptual buckets, allowing all text segments of a conceptual bucket to be shown together.

### Evidence synthesis

Having grouped several text segments by conceptual bucket and similarity, we aimed to analyze text segments looking for context-mechanism, mechanism-outcome, context-outcome, or context-mechanism-outcome linkages implied within the text, while also paying attention to potentially repeating patterns when looking at several similar text segments. We added additional columns in the analysis table to serve as analytic steps towards developing CMOCs. A simplified version is presented in Table 3 to illustrate the procedure for a central CMOC in our study. We examined the text segments for concepts and explored how the concepts were being related to each other within the text. This resulted in a note of the concept and its function within that particular text segment, i.e., whether a concept appeared to function as a context, mechanism, or outcome (Column 3). For example, in Quote 41, the concept “Formal education arrangement, informal spaces, and opportunities to socialize” appeared to be a context (C). Another concept was “Know each other on a personal level”, which could be interpreted as a mechanism (M). This particular text segment provided a potential context-mechanism linkage. Text segments with similar content were coded in the same manner. The next step (Column 4) was to further abstract the extracted concepts and potential CMOC components from Column 3. For instance, “Formal education arrangement, informal spaces, and opportunities to socialize” became “Time and space to socialize”. In a next step, we tried to fit the various abstracted concepts and their linkages into a coherent CMOC, using the original text segments as aids into a CMOC template statement in the form of: “If/when [context], then [outcome], because [mechanism].” In this process, we allowed ourselves to also be guided by the information gained from the SME interviews and MRTs extracted from the included studies and other MRTs that the research team judged potentially relevant. For instance, the idea of interprofessionally collaborating professionals liking each other being a contributing factor was indicated in one SME interview. As an MRT, Contact Hypothesis was judged to be relevant already prior to data analysis. Some CMOCs, such as the one presented here, were strongly informed by prior theory and interview data, and the data in the studies included supported them. Other CMOCs were more strongly data-driven. Having formulated a candidate CMOC, we compared its fit with other text segments. In some CMOCs, amendments were made to the structure of the CMOC, e.g. what appeared to be an outcome was sometimes discovered to be more suitably interpreted as, for instance, a mechnanism for our research question. For example, “knowing each other professionally and personally” might be considered an outcome of IPE, but it seemed more appropriate to view it as a mechanism which leads to the outcome “trust, respect, and liking among learners”. When we were satisfied with the fit between candidate CMOC and validation text segments, we considered them as pre-final CMOCs. To validate the CMOCs, MRTs from psychology were explored that could explain the underlying mechanisms we extracted from the data, as MRTs offer a more generalized theoretical explanation than a CMOC (Wong et al., 2010). We selected theories that were capable of explaining various phenomena related to positive attitude development and which helped to make explicit theoretical links (Hean et al., 2016) between IPE, intermediate variables, and attitudinal outcomes. Where possible, we selected theories that were previously applied in IPE research, as previous use provides additional evidence of the theory’s applicability for IPE research. The refined PT and CMOCs based on data from included review documents were shared with SMEs for feedback, to ensure rigor and relevance for IP educators and practitioners, and to garner recommendations for enhancements to current IPE.

**Table 3:** CMOC development

Reference	Quote	Concepts and CMOC Components	Abstraction	CMOC
Quote 41 Friman et al., (2017, p. 624)	The formal education arrangements also created informal spaces and opportunities to socialise and get to know each other on a personal level	Formal education arrangement, informal spaces, and opportunities to socialize (C) Know each other on a personal level (M)	Time and space to socialize (C) Knowing each other personally (M)	When IPE provides time and facilities for formal and informal interactions (C), it fosters trust, respect, and liking among the learners and helps to reduce professional stereotypes (O) because it allows them to get to know each other professionally and personally (M)
Quote 23 Berger-Estilita et al., (2020a, p. 7)	Such interventions allow for exchange of knowledge or skills and sharing of different experiences, which improves understanding and communication between groups, and builds trust	Sharing of different experiences (M) Improves understanding and communication (O) Builds trust (O)	Knowing each other professionally (M) Respect (O) Trust (O)	
Quote 7 Berger-Estilita et al., (2020a, p. 15)	The social component of IPE was mentioned as a goal and as an advantage. Students considered the networking beneficial, and by engaging on interprofessional relationships on a personal level, they could learn about each other’s curricula in informal settings and even foster friendships	Social component of IPE (C) Engaging on a personal level (M) Foster friendships (O)	Interaction (C) Knowing each other personally (M) Liking one another (O)	
Quote 77 Snyman and Donald (2019, p. 331)	Students suggested that being in relationship with people from other health professions outside of professional context – be it family members, friendships, romantic relationships or shared living spaces – had a valuable influence on their increased knowledge and positive perception of other health professionals	Relationship with people from other health professions outside of professional context (C) Increased knowledge (M) Positive perception of other health professionals (O)	Informal interaction (C) Knowing the other personally (M) Liking/positive attitude towards other health professionals (O)	

Context, mechanism and outcome denoted by corresponding capitalized letter in parenthesis. The full data set of quotes are featured in Supplementary File 2 and referenced by Quote Number

We constructed six CMOCs to reflect the outcomes described in the studies and to describe the theoretical links proposed by the developed PT model, the development of which is described in the following chapter.

## Model building

**Fig. 2:**
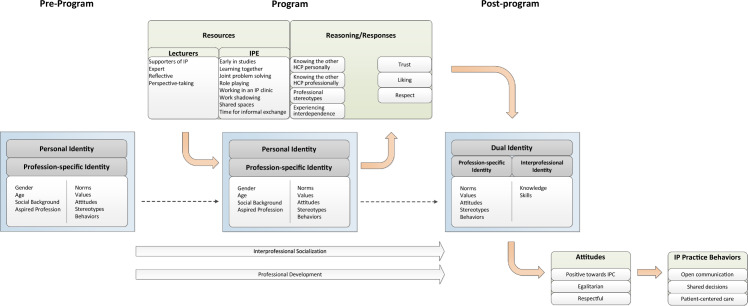
Program theory

As part of IPT and PT development we built a model to describe the components and processes of a learner’s interaction with IPE and their development within it. Its basic structure is based on Biggs’s (1987, 1993) 3P model, which features a three-phase model of student learning, comprising the phases of presage, process and product. It was also used as analytical tool in two systematic reviews on the effects of IPE (Hammick et al., 2007; Reeves et al., 2016), demonstrating its utility in modeling IPE as a process and describing its components. In an analysis iteration we relabeled the phases to “pre-program”, “program”, and “post-program” to better align with presenting IPE as a program of teaching and learning that extends beyond any one course or educational or healthcare institution. The model is a schematic representation in which we integrated theoretical concepts and propositions derived from the preliminary literature review, SME qualitative interviews and conceptualizations of identity, socialization, and development as they are commonly applied in sociological, psychological, and education literature (e.g., Haller & Müller, 2008; Hurrelmann, 2002; Oerter & Montada, 2002). The model helped inform our realist synthesis search strategy and data extraction (e.g., conceptual buckets). We revised the model iteratively as indicated by new data and analyses from the systematic literature review and CMOC development. The final model is featured in Fig. 2.

## Results

Our study results are presented in the sequence of realist theory development, beginning with the IPT and followed by the refined PT. We present the characteristics of the 23 studies included in the realist review in Table 4. Realist review exemplar quotes are provided in Supplementary File 2.

**Table 4:** Extracted data from included studies

Study	Design	Study Objective	Sample and Setting	IPE Characteristics	Putative Contextual Factors	Putative Mechanism	Outcomes of Interest
Berger-Estilita et al. (2020a)	MM	Determine whether there are changes in attitudes towards interprofessionalism between the bachelor’s and master’s program Establish which time in the medical curriculum is ideal to introduce IPE	Medical students University of Bern, Switzerland	Two optional interprofessional internships One compulsory seminar on confidentiality (medical, law, nursing students) One compulsory intravenous canulation workshop with groups of up to 6 students (medicine, nursing, midwifery, technical professions)	Some medical students had discriminating attitudes towards nursing students Most medical students were unhappy about knowing less than the nursing students Groups need to be deliberately mixed to avoid same-profession groups Knowledge imbalance among students: only some students can benefit / provide support Poorly structured course Having overloaded schedules Fear of losses: loss of professional (medical) identity, loss of specialization, loss of thoroughness in the curriculum, superficial treatment of topics Lack of assessment (grade) A poor quality IPE experience Characteristics of other participants Balancing the right amount of IPE	IPE enables the exchange of knowledge or skills and sharing of experiences This “improves understanding and communication between groups, and builds trust” (p. 7) Teaching and practice with a skilled practitioner Relaxed, informal interaction Information exchange and guidance from (already) knowledgeable nursing students Recognizing and valuing what the other professions have to offer Finding things in common intensifies social relationships at work and outside of work Viewing IPE as necessary vs. as secondary, superfluous, less relevant, unworthy Enhancement of prejudices	Change in attitudes as measured with G-IPAS Women showed higher attitude scores Bachelor (pre-clinical) students had higher attitude scores compared to master (clinical) students
Berger-Estilita et al. (2020b)	RV	Determine optimal time to introduce IPE in the medical curriculum	Various countries and universities (N = 23 studies) Medical students	Various interventions	Country/setting Public/private university Cohort/class size	Early vs. late IPE introduction	Attitudes towards IPE/IPC and other professions
Biehle et al. (2019)	QN	Evaluate pharmacy students’ perceptions of other HCPs’ characteristics after IPE	USA, University of Wyoming, School of Pharmacy Pharmacy students	Internal medicine advanced pharmacy practice experience (8-week rotation) Multi-day intervention Interactive small-group learning activities and faculty presentations	Timing of IPE Exposure to other professions	Pre-existing stereotypes influence judgments of one’s own and other professions Contact with professionals from other professions leads to a correction of their judgments post-IPE	Student ratings of pharmacists’ characteristics (SSRQ) Student ratings of other HCPs’ characteristics (SSRQ)
Bloomfield et al. (2021)	QN	Evaluate the effect of a large-scale IP workshop on attitudes towards IP socialization	Australia, University of Sydney, Faculty of Medicine and Health Medical, nursing, pharmacy students in first year of graduate degree	Students randomly allocated into mixed groups of 6–8 students 3-h workshop with three activity stations, focusing on teamwork, communication skills and HCP roles Students work together to complete activity at each station	Discipline Having an important assessment close to the workshop date Personality of other students and feeling intimidated to speak up	Learning more about the roles of one’s own and other professions Dispelling or reinforcing stereotypes Opportunity to engage in social interaction Working together on a task Communicating with each other	Attitudes (ISVS-9, short form) Significant increase in attitude scores for nursing and pharmacy students No significant change in attitude scores for medical students
Filies and Frantz (2021)	QN	Assess first- and final-year students’ attitudes towards IPC	South Africa, University of Western Cape First- and final-year students Multiple disciplines (e.g. dentistry, dietetics, natural medicine, nursing, pharmacy, etc.)	Primary health care module No specific course, course content or teaching method described	Gender Discipline Year of study (freshman vs. senior year)	Attitudes improve across time as students gain more experience of healthcare services and learn about IPC IPE produces positive affect	Attitudes towards IPC (measured with an adapted version of RIPLS) Attitudes: on average better among seniors vs. freshmen No significant attitude change among students of certain disciplines (e.g. dietetics, occupational therapy, pharmacy) Female students developed more positive attitudes because IPE focuses on a communication style that reinforces women’s preferences for relationship-building
Friman et al. (2017)	MM	Investigate nursing and medical students’ attitudes towards each other’s future professions and IPC	Sweden, Karolinska Institute Second-year nursing and third-year medical students	IP learning activity on wound care Two skills training sessions One session of patient case analysis and reflection on professional identity and stereotypes	Discipline: nursing or medicine Education arrangements that provide informal spaces to socialise Different learning climates in nursing/medicine (nursing more collaborative; medicine more independent and competitive)	IPE increases awareness of a student’s own attitudes IPE allows students to know the other profession’s knowledge and skills IPE helps students become more comfortable in their future roles IPE can increase awareness of roles	Attitude scores on the Jefferson Scale of Attitudes towards Physician-Nurse Collaboration Scores post- vs. pre-IPE non-significant Higher attitude scores among nursing students pre- and post-IPE
Fusco and Foltz-Ramos (2018)	QN	Evaluate the effect of simulation-based learning on pharmacy and nursing students’ perceptions of IP care	USA, University at Buffalo, Jacobs School of Medicine and Biomedical Sciences Third-year pharmacy and final-year nursing students	High-fidelity simulation-based learning based on two-hospital based scenarios Simulation-based learning session is followed up by a debriefing	High pre-IPE attitude scores Discipline: nursing vs. pharmacy Nursing students had higher pre-IPE scores than pharmacy students, which may be explained by their having had more clinical experience and experience working with other HCPs	When pre-IPE attitude scores are already high, high-fidelity simulation-based learning can lead to even higher attitude scores post-IPE	Attitudes as measured with the Student Perceptions of Interprofessional Clinical Education-Revised (SPICE-R) Attitudes improved in all items among pharmacy students and 9 out of 10 items among nursing students Nursing students had significantly higher scores than pharmacy students in 7 out of 10 items
King and Violato (2021)	QN	Assess HC students’ attitudes toward IPE/IPC over a three-year period	Canada, University of Alberta Students from 13 different health science programs (e.g., medicine and dentistry, rehabilitation medicine, nursing, physical education, pharmacy, etc.)	Experiential Learning Theory-based IPE Integration in all 2- and 4-year programs Begins in the first weeks of study First session: 3-h experiential learning 3-credit IPE course required for all students Additional IPE requirements and optional courses vary by program	Faculty (discipline) Cohort	Taking more IPE courses contributes to an improvement in attitudes towards IPE/IPC Higher attitude scores are expected as the students progress through their studies Attitudes and attitude improvement may differ by faculty and cohort	Attitudes towards IPE/IPC as measured with IPAS No significant differences year-to-year in any of the IPAS subscales Only small, statistically significant but non-meaningful differences by cohort in the patient-centredness subscales Exposure to an IP course only associated with patient-centredness subscale Other previous exposure to IPE and number of years of university not associated with higher IP attitude scores
Lockeman et al. (2017)	QN	To investigate whether simulation-based IPE promotes change in attitudes and stereotypes	USA, Virginia Commonwealth University Final-year nursing Bachelor students, fourth-year medical students	Two-week interprofessional simulation-based education program Featured 3 two-hour simulations over two weeks focused on acutely ill patients Students grouped into IP teams of 6–7 members	Discipline: nursing vs. medical students	Improves attitudes towards IPC by influencing professional stereotypes	Attitudes towards IPC as measured with SPICE-R2 Perception of stereotypes as measured with the Healthcare Stereotypes Scale Attitude scores increased post-IPE Positive attributions (i.e., positive stereotypes) toward nurses increased, but not toward physicians
Matulewicz et al. (2020)	MM	To explore attitudes and learning outcomes among early-level health profession students	USA, Virginia Commonwealth University Undergraduate and doctoral health profession students: e.g., medicine, nursing, pharmacy, occupational therapy	2 one-credit, semester-long, foundational IPE courses 500 students divided into IP groups of 6 members each	Discipline	IPE shapes professional identity development, but differentially by discipline Students normally acculturated in an environment focusing on individual performance IPE affects attitudes by refocusing performance toward patient-centred care, in which all professions contribute	Attitudes towards IPC as measured with SPICE-R2 Student attitudes generally more positive post-IPE Pharmacy students showed highest increase in attitude scores
McGregor et al. (2018)	QN	To identify dental and dental hygiene students’ attitudes towards IPE after attending a novel IPE course	USA, Virginia Commonwealth University Students from 6 healthcare professions, e.g., dentistry, nursing, pharmacy	13 one-hour session course targeting 3 IPEC competency domains Learning objectives: knowledge of IPC and professional roles, develop team-based skills	Discipline	IPE has an impact on student attitudes which differs by profession Students from professions that see themselves as collaborative practitioners (e.g., dental hygiene students) are more likely to experience an increase in attitude scores than students from professions that see themselves as leaders of the healthcare team (e.g., dentistry students)	Attitudes towards IPC as measured with SPICE-R2 Student attitudes increased post-IPE No significant differences between dental and dental hygiene students were found Dental hygiene students had the greatest increase in attitude scores
Mowat et al. (2017)	MM	To examine whether a continuing education IP program improved participants’ attitudes towards IPE and collaboration between dental and non-dental HCPs	Canada, University of Manitoba, Rady Faculty of Health Sciences and Office of Continuing Competency and Assessment of the Colleges of Medicine and Dentistry Participants included: physicians, dentists, dental hygienists, nurses, etc.	IPE event: “Oral-Systemic Health Day” Included lectures, roundtable discussions of two case studies, and an optional oral cancer screening workshop	Discipline Workplace culture where IP is practiced	Complex case studies allow different HCPs to come together to share personal and professional perspectives The experience is perceived as rewarding and worthwhile leading to improved attitudes Workplace culture may have a strong effect on attitudes and cause them to revert to baseline after enough time has elapsed There may be latent beliefs among physicians that they have a privileged role in care, which may contribute to lower attitude scores compared to other professions	Attitudes towards IPC as measured with RIPLS Attitude scores increased post-IPE Differences between professions observed: significantly lower scores among physicians compared with nursing and dental hygiene professionals Scores returned to baseline 6 months after the IPE event
Muzyk et al. (2019)	QN	To evaluate an IPE course on student educational outcomes attitudes towards IPC	USA, Duke University Health System Students attending 1-month psychiatry clerkship Student disciplines: medicine, nursing, physician assistant, pharmacy, social work	IPE course on substance use disorder Composed of 4 interactive class sessions, 1 recovery meeting, 1 short reflection paper Optional: conduct a counseling session with a patient	Discipline	IPE specifically grounded in Mezirow’s Transformative Learning Theory Creating a learning environment that encouraged active participation, learning of new roles and perspectives, role play and role modelling The IPE environment allowed students to share their learning experiences, beliefs, personal stories, which is experienced as positive and rewarding, leading to improved attitudes towards IPC	Attitudes towards IPC as measured with SPICE-R2 Attitude scores increased post-IPE No significant differences in attitude scores found between medical and non-medical students
Ng et al. (2021)	QL	To examine changes in attitudes or practice after completion of an IP clinic placement	Canada, Toronto, Interprofessional Medical and Allied Groups for Improving Neighborhood Environment, a student-run interprofessional clinic 23 healthcare students volunteering for clinical shifts in a student-run free clinic Students of medicine, nursing, pharmacy, physiotherapy, and social work	3-week consecutive clinical shift Includes a mandatory reflection session	Seeing underprivileged patients in a more positive light Positive interactions with other HCPs	Attitude change is triggered by the authentic experiences students make as practitioners in a free clinic Students meet with people from underprivileged groups in the population Students see how collaboration with other HCPs is necessary This leads to a shift in thinking and a valuing of IPC Transformative Learning Theory describes how a shift in thinking occurs when one’s views is challenged by the situation (so-called disorienting dilemmas) Attitude change facilitated by questioning own assumptions (because of experiences made in the practice setting), authentic interactions with other HCPs and patients, and seeing themselves as part of an interprofessional team	Attitude changes and practice changes More egalitarian view of marginalised populations
Roberts et al. (2018)	QN	To examine the links between professional identification, communication/teamwork skills, perceived relevance of IPE, and positive/negative attitudes towards IPE	Australia, Curtin University, Faculty of Health Sciences Students from 25 health science professions, including nursing, midwifery, speech pathology, occupational therapy, physiotherapy, etc.	First-semester IPE curriculum consisting of 5 compulsory core units, 2 common units completed in smaller groups, and 2 profession-specific units	Large class sizes vs. small face-to-face IPE Representation of each healthcare profession in the case being discussed in class Preventing a skew of the number of class participants towards one dominant profession Self-reported communication and teamwork skills	When IPE courses are designed to make the relevance of IPE in clinical practice more salient, students will believe IPE to be more relevant to their future careers and therefore, the students are more likely to develop positive attitudes towards IPE	Positive attitudes towards IPE as measured with the 9-item Interprofessional Learning subscale (Pollard et al. 2004) Negative attitudes towards IPE as measured with an 8-item scale (Roberts and Forman, 2014) 4-item perceived relevance of IPE scale (Roberts and Forman, 2014) Perceived relevance mediated the link between professional identification and positive/negative attitudes towards IPE
Seaman et al. (2018)	QN	To examine students’ beliefs, behaviors, and attitudes and how they relate to interprofessional socialization, expectations, and previous tertiary education experience	Institution not indicated in the study description, but presumably University of Western Australia, based on ethics statement Final-year master of nursing science and bachelor of medical science/bachelor of surgery students	2-week ambulatory clinical placement Students divided into paired interprofessional teams and placed in two of four clinical outpatient areas, collaborating with senior clinical staff from their own and from other professions Outpatient areas include clinics in respiratory medicine, diabetes and endocrinology, pain	Previous tertiary education Previous experience in health	Placements are important for developing IPC understanding and an IPC identity	Attitudes as measured with an adapted version of the Interprofessional Socialization and Valuing Scale (ISVS) Positive change in attitudes and behaviour after a clinical placement Attitude score increases higher among nursing students than medical students
Skolka et al. (2020)	MM	To analyse student responses to IPE	USA, Penn State University College of Medicine First/second year medical and physical assistant students; third/fourth year nursing students	Interprofessional clinical immersion experience, serving in a medical brigade in Central America Students provided medical care in 1-week outreach for underserved population Students supervised in their clinical practice by physicians, physician assistants, dentists, pharmacists and supported by an interpreter Students rotated through different stations Reflection/debriefing sessions conducted in the evenings Students began preparation 3 months ahead of deployment	Level of clinical and interprofessional experience Profession Profession correlated with clinical experience, with physician assistants having the most experience Medical students had the least prior clinical experience Difficulties in interpersonal relations Familiarity with team members	When idealistic students with highly positive attitudes towards IPE collaborated with students from other disciplines and schools that were unfamiliar to them, they came to realize that interprofessional collaboration is not always easy, due to interpersonal dynamics, conflicts, and misunderstandings IPC is easier when team members know each other well. IP clinical immersion provides the opportunity to get to know other team members and to quickly learn to collaborate effectively	Attitudes towards IPC as measured with RIPLS No significant changes in attitudes overall, but small non-significant decreases measured in attitude subscales including professional identity, teamwork, and collaboration Patient-centredness motivated students to work through team conflicts and find the best ways of performing tasks
Smith et al. (2020)	MM	To determine if interprofessional point-of-care ultrasound (POCUS) training changed resident physicians’ attitudes towards IPE and sonographers	USA, a Midwestern university for health professions Diagnostic medical sonography students teaching internal medicine residents point-of-care ultrasound	IPE workshop content: diagnostic medical sonography (DSM) students are to teach internal medicine residents (IMR) point-of-care ultrasound DSM students first trained in 2-h train-the-trainer workshop consisting of 4 scripted simulation-based teaching scenarios In a 3-h IPE workshop, DSM students teach IMRs POCUS exams of the kidney, bladder, and aorta IPE workshop informed by the Contact Hypothesis	Both groups of participants (teachers and learners) both still in training, creating a learning environment perceived as safe Students of a profession considered lower-status taught professionals of a profession considered higher-status As they are DSM students, having profound enough knowledge to teach may be perceived as impressive by the IMR learners IMRs were taught a skill they need in their profession which is also a topic popular among trainees Both groups of participants had little interaction with the other profession prior to the IPE	IPE provided the opportunity for contact with other professionals and learn about one another’s background in a safe environment This contributes to increased appreciation of each other’s skills and contributions The increased appreciation leads to improved attitudes This is in line with the Contact Hypothesis, which states that contact with a specific group of people can increase positive attitudes towards that group	IMR attitudes towards IPE as measured with 9 adapted items from RIPLS and 5 questions from an IPE scale developed by Gardner (2002) IMR attitudes towards sonographers as measured with the Student Stereotypes Rating Questionnaire (SSRQ) IMR IPE attitude scores and attitudes towards sonographers improved post-IPE DMS also benefited from the IPE in terms of having more confidence in communicating with physicians
Snyman and Donald (2019)	QL	To determine how an early interprofessional service-learning initiative influenced students’ interprofessional practice	South Africa, Stellenbosch University Medical, dietetics, and occupational therapy students	4-phase service-learning curriculum Phase 1: assess the needs of the community related to childhood development and parenting Phase 2: plan and develop an intervention with providers and users Phase 3: implement the intervention, e.g., parenting workshops, home visits, etc Phase 4: iteratively improve the intervention Medical students had additional task completing case study of two patients and serve as their case managers, while consulting with and drawing on the expertise of the occupational therapy and dietetic students	Discipline/aspired profession: different disciplines exposed to different learning environments prior to and during the IPE leading to differences in exposure to holistic patient management and perceptions of the IPE (rural placement) Attitudes pre-IPE Learner’s perceptions, values, beliefs Length of time spent on site: shorter visits led to less opportunity for collaborative and informal learning Favouring profession-specific training Viewing the IPE (service-learning activity) as detracting from other important learning Considering the IPE experience as valuable	The IPE was intended to provide support in healthcare to help in parenting and healthy child development using medical, dietetics, and occupational therapy students, who were brought together to develop and implement an intervention to support these objectives (i.e., develop a parent support program) The IPE provided students the opportunity to experience the importance and value of IPC This is especially true for students who have only experienced hospital settings that were hostile to IPC and were therefore unaware of the value of IPC before seeing it in action for themselves Existing attitudes shaped the effect of IPC on attitudes, as well as any previous experiences and exposure to IPC Having personal relationships with professionals from other professions can help to support the positive impact of IPE (e.g., having a family member, living with, or having a romantic relationship with a professional from another healthcare profession)	Attitude towards IPC Effects of IPE on attitudes varied by discipline, existing attitudes towards IPC and other health professions Students with positive attitudes did not feel that their attitudes changed, i.e., they maintained their positive attitudes Students that had personal relationships with professionals from other professions reported increased knowledge and positive attitudes
Squires et al. (2021)	QL	To understand how previous work and educational experiences inform students’ attitudes towards IPE/IPC	USA, presumably New York University, Rory Meyers College of Nursing (not explicitly stated), and affiliated medical centre, and community-based home care agency Secondary data analysis of 10 focus groups and 10 individual interviews with a total of 75 participating health profession graduate students (nurse practitioner, social work, midwifery, and primary care internal medicine students) Students taken from 2 IPE programmes	Programme A: medication management intervention to reduce medication complexity among older adults; with nursing, social work, and pharmacy graduate students Programme B: intensive intervention to enhance geriatric care quality; with nurse practitioners, primary care medical residents	Physician participants reported having had no undergraduate IPE, resulting in gaps in medical education in terms of IPE Physician participants least likely to have any previous work experience, having directly transitioned from higher education to medical school Most participants with work experience report having participated in voluntary IPE, some offered (but not required) by their employer Participants who participated in IPE as continuing education did so voluntarily Some participants experienced IPE as part of mandatory on-the-job training (e.g., the TeamSTEPPS programme), for instance for substance abuse management Workplace attitudes towards IPC may vary by location of the healthcare workplace (for instance, rural vs. urban) Rural vs. urban location where student experienced/experiences IPC may be linked with organisational culture towards IPC A hospital with more financial and staffing resources may have a culture more supportive of IPC Some specialties may have a stronger orientation towards IPC, e.g., oncology, pediatrics	Previous experience with IPE and IPC shapes a learner’s perceptions/attitudes of IPE and IPC Depending on whether the learner has previously experienced IPE and IPC, the amount experienced and the quality experienced can differentially shape a learner’s attitudes Because medical residents have had less experience with IPE and IPC during their studies and residency, they may be more reserved or may tend to have more negative attitudes towards IPE and IPC compared with learners from other healthcare professions Nurses tend to have had more experience with IPE and IPC, which leads to more opportunities of making positive experiences with IPE and IPC, making their attitudes generally more positive Nurses may still develop negative attitudes or be less convinced of the value of IPC if they have made negative experiences with IPC in their workplace, for instance being belittled or ridiculed by attending physicians when making suggestions or speaking up If workplace culture is unsupportive of IPE/IPC, workers will adopt the organisation’s norms and work less collaboratively	Perceptions of interprofessional collaboration The perceptions include value-laden perceptions, i.e., positive and negative perceptions of IPE/IPC These perceptions can be considered attitudes, as they are an evaluation of an object or a situation Learners who have had positive experiences with IPC spoke with stronger conviction of IPC’s value in healthcare delivery Learners with mixed or negative experiences did not express such convictions Positive attitudes towards IPE and IPC were associated with having had previous experience of IPE and IPC, having had positive experiences of IPE and IPC. This provides an explanation of the link between profession and attitudes towards IPE and IPC Organisational culture that is supportive of IPC may be associated with more positive experiences of staff with IPC and therefore more positive attitudes
Stephens & Ormandy (2018)	QL	To evaluate the impact of an IPE program on the development of students’ affective domain learning (i.e., impact on attitudes, values, and behavior)	UK, presumably University of Salford (authors’ affiliation), School of Nursing, Midwifery, Social Work and Social Science third-year, pre-registration students of nursing, physiotherapy, podiatry, radiography, and social work Students allocated to one of three NHS sites	Program focused on exploration of communication, team building, therapeutic relationships, and clinical and professional skills Delivered over a 6-week period Targeted learning outcomes: IP knowledge, attitudes, skills	Having a practice setting important: students gain a professional identity in the practice setting, while having a student identity in the university setting Supports having a more “professional mindset” May support in having positive attitudes and values towards IPC Feeling being treated as equals by facilitators How far along they are in their studies helps them feel more confident in their interactions with their patients and other professionals Whether attitudes will translate into corresponding behavior depends on the social norms of the work environment This explains how despite a change in attitudes, behavior does not always follow	IPE affects attitudes by: Allowing students to experience what good collaborative practice is by: o Seeing what was taught in theory come alive o Being able to appreciate the roles of other professions o Seeing/understanding team dynamics and what makes teams work well o Having the space to discuss experiences from practice Enabling professional identity development by: o Having satisfying relationships within the group o Developing better self-awareness o Changing their views through insight into other professions’ roles o Improving students’ confidence o Having a professional identity in the practice setting (as opposed to a student identity) o Positive feedback loop affects attitudes, values, behaviors Providing learning alliances that: o Enable students to learn with, from and about each other o Foster good relations between students and facilitators o Enable positive group dynamics and support flat hierarchies o Allowed students to learn about different team roles and identify their own role within the team Providing circles of care which: o Includes members of IP teams to replicate the practice setting o Include a broad representation of different professions o Enables gaining appreciation and developing respect for other professions Sustained changes are achieved by internalising attitudes into values, i.e., progressing to Stage 3 in the Epstein model Repeated or longitudinal IPE is a requirement for attitude change Real clinical situations, repeated over time, giving opportunity to reflect has an impact on attitudes, values, and behavior	Positive effects on attitudes, values, and behavior Transitioning from holding only holding positive attitudes in conditions of group surveillance (Epstein’s Developmental Level 1)or to satisfy personal relationships (Level 2), to an internalisation of the attitudes such that they are seen as congruent with their own values (Level 3)
Thompson et al. (2020)	MM	To investigate nursing and medical students’ readiness for IPE To define the optimal number of IPE sessions	UK, Oxford, John Radcliffe Hospital, Oxford University Medical School, Oxford Brookes University Second-year nursing students, Fourth-year medical students	PBL-based IPE featuring standardized cases relevant to geriatric practice Workshops with 30–45 min. introduction, 2 h self-directed learning, and facilitated session with nurses and geriatricians Facilitated sessions aimed to improve professional socialization Students encouraged to discuss cases from their professions perspective Intervention group: medical students paired with nursing students in groups of maximum 10 students Control group: medical students who were not paired with nursing students; had discussions only with facilitators	Nursing and medical students may have different concerns regarding IPE Nursing students concerned with being seen as inferior and not being taken seriously by medical students Medical students concerned with being perceived as arrogant, pretentious, or condescending by nursing students A welcoming, respectful, relaxed session may contribute to more openly expressing opinions in IPE discussions Having members of another profession present in IPE may not be necessary for IPE to have a positive impact on attitudes; the role of the other profession in group discussions can be taken up by the facilitators 10 students per group might be the maximum group size for optimal in-group discussions	Enjoyment of the activity with the other profession Learning about the other profession Nursing: Boosting confidence of nursing students, decreasing concerns and feelings about being seen as inferior or not being taken seriously Medical students: gaining a better understanding of the nurses’ perspective, gaining appreciation of the nurses’ role IPE helped students to learn about and appreciate the role of the other profession and alleviate preconceived fears regarding interactions with students/members of the other profession	Attitudes towards IPC as measured with RIPLS and open-ended questions Attitudes improved among nursing and medical students Attitudes also improved in the medical students in the control group Nursing students in the intervention group improved in all four RIPLS subscales Medical students in the intervention group improved in two of four subscales (teamwork and collaboration, positive professional identity) Medical students in the control group have the same outcome and improved in the same two subscales
Yang et al. (2017)	QN	To evaluate whether benchmarking can successfully cultivate seed instructors who can improve team members’ attitudes towards IPC	Taiwan, Taipei Veterans General Hospital Physicians, nurses, and pharmacists with more than one year but less than four years of clinical experience	3-month IPE intervention using a debrief diamond model-based IPE simulation Participant attitudes measured during three-month period: before IPE, after IPE, and at end of study Participants attended a 3.5-h preparation course in the first study month (T_1_) In the second month (T_2_), participants attended a 3.5-h simulation course In the final month (T_3_), half of the participants were randomly assigned to participate in the post-course IPC benchmarking, where there transfer of skills into practice was rated by facilitators, using the debrief diamond	Pharmacists and nurses had more frequent exposure to IPE than physicians Providing access to an IPE e-learning platform may improve attitudes and help maintain them by providing continuous engagement with IPE Lower attitude scores among physicians compared to nurses and pharmacists are maintained even post-IPE, despite an increase in scores among physicians	Higher attitude scores are possibly caused by improving interprofessional relationships, communication skills, efficiency of care delivery, teamwork, mutual respect, and confidence When participants become seed instructors and take their newly acquired knowledge into the workplace and practice what they learned, it may improve attitudes towards IPC and IPC in the workplace and reinforce the seed instructors’ positive attitudes towards IPC, as they are now role models for the other staff Benchmarking may invite critical self-comparisons to others and critical self-reflection and encourage upholding high professional standards, which in turn promotes a higher estimation of IPC	Attitudes towards IPE, IPC, and healthcare teams as measured by the Interdisciplinary Education Perception Scale (IEPS) and the Attitudes Towards Healthcare Teams Scale (ATHCTS) Physicians had lower baseline attitude scores in the IEPS subscales ‘competency and autonomy’ and ‘understanding others’ values’ compared to nurses Pharmacists had lower baseline attitude scores in the IEPS subscale ‘competency and autonomy’ and ‘perception of actual cooperation’ compared to nurses Post-IPE: ‘Competency and autonomy’ scores of the IEPS subscale and ‘team efficiency’ scores of the ATHCTS subscale increased in all professions Score increases greater among nurses and pharmacists than physicians

### Initial program theory

The development of the IPT combined findings from the preliminary literature review and SME qualitative interviews. We used Biggs’s (1987, 1993) Presage-Process–Product (3P) framework (1987, 1993) cited in two systematic reviews on IPE (Hammick et al., 2007; Reeves et al., 2016). We renamed the 3Ps as into the phases “Pre-Program”, “Program”, and “Post-Program”, which together comprise the overarching structure of the PT model (see Fig. 2). In the Biggs framework, presage and process factors are hypothesized to interact to produce the outcome. “Presage” encompasses the conditions prior to IPE and include the characteristics of teachers and learners as well as the “sociopolitical context” (Reeves et al., 2016, p. 658). These provide the context within which the mechanisms of IPE operate (Hammick et al., 2007; Pawson & Tilley, 1997). “Process” describes the IPE approaches to teaching and learning that were applied (Hammick et al., 2007; Reeves et al., 2016) which are factors hypothesized to interact to affect attitudes.

In addition to the preliminary literature, interviews provided some additional key concepts to include as conceptual buckets during data extraction. “Trust,” “respect,” and “liking” were repeatedly used by SMEs to indicate positive conditions for IPE and IPC. For example:

I think interprofessional collaboration is very much based on knowing (…) who has what skills. (…)that requires that there is sufficient contact between the different professions already during training(…) so that you have sufficient trust (…) (Quote 46, Interview 2).

This means that if people like each other, then everything works well on its own. (…) And when people know each other as friends and know personal things [about each other], that creates trust. (Quote 12, Interview 4)

SMEs also noted how some professions may be less receptive to IPE and IPC:

We have been able to determine a clear, clear difference among the students (…) that [interprofessional] collaboration is also viewed as more important by nurses. So, there are differences early on. Quote 33, Interview 3)

What has always come up as a difficult professional category for interprofessional collaboration (…) [is] with physicians. (…) we have seen what the setting can do. (…) [Settings] where [students] have to master everyday situations together. And [students] realise that you really have [to work] together. (Quote 32, Interview 3)

Based on our preliminary search and SME interviews, we summarize the IPT as follows: IPE needs to provide the circumstances that allow enough contact between learners, so that they get to know each other as professionals and also personally. Through IPE, trust, respect, and liking among learners generate positive attitudes when  they realize that they share common values and goals, need each other to solve common practice problems, and must work together to successfully deliver care in complex healthcare settings. Even though there may be differences among learners aspiring to different healthcare professions, developing positive attitudes can be achieved among all professions by fostering sufficient contact and providing a learning setting where learners experience interdependency and collaboration among different professions.

### Refined program theory and causal context-mechanism-outcome explanations

Our refined program theory is schematically presented in Fig. 2, beginning before IPE (pre-program), continuing through IPE (program) and concluding with the completion of the program (post-program). This is an adaptation of the Presage-Process–Product or 3-P model (Biggs, 1987, 1993; Hammick et al., 2007; Reeves et al., 2016). The following sections provide overviews of each phase of our developed model in relation to evidence from our realist synthesis.

Table 4 summarizes key data extracted from the 23 papers included in our realist synthesis.

Table 5 summarizes the CMOCs associated with the refined PT (Fig. 2) and a few exemplar quotes are provided for each CMOC. Supplementary File 2 provides additional supporting quotes for the CMOCs.

**Table 5:** CMOCs and associated quotes and MRTs

Phase	CMOC	Quotes	References	MRT
Pre-Program	CMOC 1: When learners have initial, discipline-specific attitude differences (C), it will influence IPE’s impact on learners’ positive attitude development (O) because learners’ existing attitudes act as anchors against which new attitudes being taught are compared (M)	Reasons opposed to an early IPE introduction included students being overwhelmed by an overloaded, integrative year; the role of “doctor” not being yet clearly defined and prejudices against other health care professions existing before medical school. On the other hand, eleven students pointed out that the IPE introduction should occur just before or during clinical years (from the third year onwards). For them, it meant a better integration of the IPE content with clinical practice, the previous acquisition of basic clinical knowledge which would facilitate the focus on the IP component, and the broader diversity of activities that could be offered. One student was concerned that such an approach would be too late to prevent the development of prejudices. Five students mentioned it was important to have IPE on a frequent, recurrent basis	Berger-Estilita et al., (2020a, p. 12)	Social Judgment Theory (Sherif & Hovland, 1961)
		The results from this review and from individual studies should be interpreted with caution: students’ educational backgrounds, as well as attitudes, expectations and stereotypes, may vary considerably between institutions and countries and may influence how the IPE interventions are experienced. This probably accounts for many differences in effectiveness of IPE activities in different settings [15]	Berger-Estilita et al., (2020b, p. 14)	
		Recent studies still report dysfunctional interactions between different professions (Hansson, Arvemo, Marklund, Gedda, & Mattsson, 2010a). This could be due to organisational deficiencies, but it could also relate to separate professional cultures nurturing negative attitudes towards other professions (Hall, 2005; Hansson et al., 2010a). Attitudes have been proposed as a key aspect in interprofessional collaboration, and these are already partly formed during education (Khalili, Orchard, Laschinger, & Farah, 2013)	Friman et al., (2017, p. 624)	
Program	CMOC 2: When IPE provides time and facilities for formal and informal interactions (C), it fosters trust, respect, and liking among the learners and helps to reduce professional stereotypes (O) because it allows them to get to know each other professionally and personally (M)	The formal education arrangements also created informal spaces and opportunities to socialise and get to know each other on a personal level	Friman et al., (2017, p. 624)	Contact Theory (Allport, 1954)
		Such interventions allow for exchange of knowledge or skills and sharing of different experiences, which improves understanding and communication between groups, and builds trust	Berger-Estilita et al., (2020a, p. 7)	
		The social component of IPE was mentioned as a goal and as an advantage. Students considered the networking beneficial, and by engaging on interprofessional relationships on a personal level, they could learn about each other’s curricula in informal settings and even foster friendships	Berger-Estilita et al., (2020a, p. 15)	
		Students suggested that being in relationship with people from other health professions outside of professional context – be it family members, friendships, romantic relationships or shared living spaces – had a valuable influence on their increased knowledge and positive perception of other health professionals	Snyman and Donald (2019, p. 331)	
	CMOC 3: When learners are put in settings where they need to work together to overcome everyday practice problems (C), they develop an interprofessional identity and learn to respect and trust each other (O) because they are able to observe how one profession can help the other, and they come to realize that they are all dependent on each other (M)	Their experiences at the clinic consolidated an appreciation that collaboration across professional boundaries can amplify any one profession’s capacity to care for patients with complex needs	Ng et al., (2021, p. 704)	Social Identity Theory (Tajfel et al., 1979)
		Practitioners develop an interprofessional professional identity as a collaborator that complements each individual’s profession-specific professional identity (Khalili et al., 2013). This theoretical goal for interprofessional educationis supported by evidence. For example, Crawford et al. (2016) demonstrated that students from other professions perceive the nursing profession differently because of interprofessional education. Studies among practitioners have shown that interprofessional education helps to redefine professional identities consistent with the dual identity model (Ateah et al., 2011; Hood et al., 2014; Langendyk et al., 2015; Meyer et al., 2015)	Lockeman et al., (2017, p. 33)	
		Also medical students stressed the need for collaboration and linked it to the complete picture of the patient. They believed that everyone’s skills would benefit by helping each other	Friman et al., (2017, p. 623)	
	CMOC 4: When IPE facilitators serve as role models to students by being experts in their field, reflective of their own practice, and cultivating feelings of equality (C), they contribute to positive attitude development (O) by displaying good behavior to emulate, eliciting positive affect in their learners, and positively shaping interprofessional relationships (M)	Unfortunately, stereotypes formed by professional interaction and societal views on professional roles are not easily modified by educational interactions alone [54]. The introduction of small-group reflections, facilitated by adequate role models, may allow students to remodel their own professional and personal attitude towards patients, to express their moral judgements from their observations of other healthcare professionals’ interactions and to share these experiences within a safe learning environment [48]	Berger-Estilita et al., (2020a, p. 14)	Social Learning Theory (Bandura & Walters, 1977)
		Professionals form identities through a process of socialization; as existing personal identities develop through communities of practice, personal and professional identities are shaped (Cruess et al., 2015). Social learning theory (Bandura, 1977) suggests that identity acquisition stems from these learning processes. The process of socialization is influenced by multiple factors, including the learning environment, peer and personal relationships, clinical and non-clinical experiences, role models and mentors, as well as formal teaching with faculty and self-assessment. …While the strength of influences for identity formation may vary among professionals, interactions and experiences can be developed by educators to help shape positive interprofessional relationships (Cruess et al., 2015)	Lockeman et al., (2017, p. 33)	
		We included students and faculty from different health professions who shared their roles and their perspectives about patient care in classroom discussions. Faculty involved all student voices in discussions and modeled collaborative approaches to patient care. We attribute the significant improvement in students’ attitudes toward interprofessionalism in all domains of assessment (unlike our previous course in which significant improvement was more limited, as noted above) to these deliberate course enhancements	Muzyk et al., (2019, p. 1798)	
		Analysis of the transcripts found that the positive group dynamics had a significant effect on the student’s values, attitudes, and beliefs about each other; that is, they felt they were being treated like a professional	Stephens and Ormandy (2018, p. 354)	
	CMOC 5: When the IPE curriculum is perceived to be career relevant, boosts confidence, and increases learners’ comfort in working with other professions in patient care delivery (C), it leads to improved attitudes (O) because learners come to expect positive experiences from IP interactions and come to value these interactions more (M)	First, in order to maintain or increase positive attitudes towards IPE in introductory programs that span professions, the curriculum needs to be designed to demonstrate relevance to the future careers of participating students from all professions. Second, as IPE may be particularly challenging for students who do not have confidence in their abilities to communicate and work effectively in teams, educators may need to focus on building these skills to decrease negative attitudes	Roberts et al., (2018, p. 39)	Expectancy-Value Theory (Wigfield & Eccles, 2000)
		(…) prior to the session concerns about learning alongside medical students; they felt intimidated and feared there would be a hierarchy, but IPE appeared to be successful in removing these concerns, with nursing students finding the sessions very open and comfortable who also indicated that they found easy to contribute to the session, and they found the group to be very welcoming and respectful, and the session to be very relaxed. The results of this study also suggest that the nursing students became more confident as a result of the teaching; with some indicating that they would be happier to approach a doctor in the future or share information with them. It would appear that IPE resulted in boosting nursing confidence around their medical peers, and decreased concerns about feelings of inferiority/intimidation	Thompson et al., (2020, p. 5)	
		Findings revealed that most students rated the workshop positively and reported that it had changed their views of other health professionals. Many commented on their enjoyment meeting and interacting with students studying health degrees different from their own, and how this had benefitted them from both a social and educational perspective	Bloomfield et al., (2021, p. 6)	
	CMOC 6: When there is organizational support for IPC and healthcare team members participate in IPE on an ongoing basis (C), sustained positive attitudes towards IPC are more likely (O) because the attitudes and values expected in IPC are continually positively reinforced, and are eventually integrated into the learners’ personal value system	It is also important to note that even if students are prepared to be collaborative when they graduate, if the organizational culture does not support IPC and operates under traditional hierarchical, non-team friendly models, then the sustainability of graduates implementing the lessons learned about IPC from these programs is threatened	Squires et al. (2020, p. 197)	Affective Domain Development (Epstein, 1977)
		The first two stages of development (compliance and identification) are types of conformity and can revert to previously held attitudes and values, as they are both extrinsically motivated and require constant reinforcement. However, the third-stage internalisation is when a student embraces the new values and they become part of their belief system (Epstein, 1977)	Stephens and Ormandy (2018, p. 349)	

The full data set of quotes are featured in Supplementary File 2

#### Pre-Program

*CMOC* 1: When learners have initial, discipline-specific attitude differences (C), it will influence IPE’s impact on learners’ positive attitude development (O) because learners’ existing attitudes act as anchors against which new attitudes being taught are compared (M).

Even before entering a healthcare programme and attending IPE, learners will differ in terms of their personal identity, e.g., gender, age, social background, and aspired profession (Fig. 2). At this time, they may already have a rudimentary profession-specific identity of their aspired profession (Roberts et al., 2018) as well as prejudices towards other professions (Berger-Estilita et al., 2020a, 2020b). This profession-specific identity can be described in terms of its norms, values, attitudes, stereotypes, and behaviors (Friman et al., 2017; Stephens & Ormandy, 2018). As attitudes may already be partly formed during education (Friman et al., 2017), these attitudes will shape how IPE is experienced and also impact IPE’s effectiveness (Berger-Estilita et al., 2020a, b; Lockeman et al., 2017). As Berger-Estilita, Fuchs, et al. (2020b, p. 14) note:

(…) students’ educational backgrounds, as well as attitudes, expectations and stereotypes, may vary considerably between institutions and countries and may influence how the IPE interventions are experienced. This probably accounts for many differences in effectiveness of IPE activities in different settings.

Several studies have shown a differantial impact of IPE on attitudes by profession (Berger-Estilita et al., 2020a, 2020b; King & Violato, 2021; Lockeman et al., 2017; Matulewicz et al., 2020; Snyman & Donald, 2019; Thompson et al., 2020). This may be partly due to individual attitude differences already present prior to training (Snyman & Donald, 2019) that select for and channel prospective students into different professions; and partly due to different professional cultures inculcated from the start of training (Friman et al., 2017). For instance, students of medicine compared to students of other healthcare professions may be more inclined to be attracted by their chosen profession’s prestige and status (Biehle et al., 2019; Friman et al., 2017) and be more individualistic, competitive, and less team-oriented (Friman et al., 2017). Similarly, students of more technically oriented professions are sometimes perceived as having poorer interpersonal skills (Smith et al., 2020). More generally, attitudes and values already present at the beginning of training will likely affect the extent to which interprofessional education can modify them (Lockeman et al., 2017).

In their study of undergraduate medical and allied health students, Snyman and Donald (2019, p. 332) conclude:

The findings suggest that students’ attitudes toward IPCP were influenced by their profession, with some professions showing less enthusiasm for IPCP than others.

#### Program

*CMOC* 2: When IPE provides time and facilities for formal and informal interactions (C), it fosters trust, respect, and liking among the learners and helps to reduce professional stereotypes (O) because it allows them to get to know each other professionally and personally (M).

IPE is described as a process of interprofessional socialization (Matulewicz et al., 2020), as it provides resources in terms of time and facilities for learners aspiring to different healthcare professions to come into contact in formal and informal interactions (Berger-Estilita et al., 2020a, 2020b; Friman et al., 2017). Friman et al., (2017, p. 624) described their IPE intervention as follows:

The formal education arrangements also created informal spaces and opportunities to socialise and get to know each other on a personal level.

This allows learners to learn about the skills, competencies, and knowledge other professions have to offer (Friman et al., 2017) and to get to know each other professionally and personally (Berger-Estilita et al., 2020a, Snyman & Donald, 2019). IPE aims to achieve IPC that is egalitarian, respectful (Bloomfield et al., 2021), and appreciative of each healthcare profession’s contributions to patient-centred care by building upon a common curriculum delivered early in a healthcare professional’s training (Berger-Estilita et al., 2020a, 2020b; Biehle et al., 2019; Fusco & Foltz-Ramos, 2018). The interpersonal relations and mutual respect developed help the learners to communicate and function better as a healthcare team (Skolka et al., 2020).

Such interventions allow for exchange of knowledge or skills and sharing of different experiences, which improves understanding and communication between groups, and builds trust (Berger-Estilita et al., 2020a, p. 7).

Getting to know the the other learners means having the opportunity to better understand what each professional’s role in care is (Fusco & Foltz-Ramos, 2018), thereby also supporting professional identity development (Stephens & Ormandy, 2018). At the same time, learning together allows professional stereotypes to be deconstructed (Bloomfield et al., 2021; Matulewicz et al., 2020) and be replaced by actual knowledge of what competencies other professionals offer and their added value in healthcare (Smith et al., 2020).

The social component of IPE was mentioned as a goal and as an advantage. Students considered the networking beneficial, and by engaging on interprofessional relationships on a personal level, they could learn about each other’s curricula in informal settings and even foster friendships. (Berger-Estilita et al., 2020a, p. 15)

Some of the advantages of IPE lie in the knowledge gained of the other professions as well as the social and relational aspects, which allow mutual trust and respect to develop. The literature’s suggestion that personal relationships and friendships have a positive impact on attitudes and that IPE may foster the development of friendships suggests liking the other as an important factor in developing positive attitudes towards IPC and other professionals:

Students suggested that being in relationship with people from other health professions outside of professional context – be it family members, friendships, romantic relationships or shared living spaces – had a valuable influence on their increased knowledge and positive perception of other health professionals. (Snyman & Donald, 2019, p. 331)

*CMOC* 3: When learners are put in settings where they need to work together to overcome everyday practice problems (C), they develop an interprofessional identity and learn to respect and trust each other (O) because they are able to observe how one profession can help the other, and they come to realize that they are all dependent on each other (M).

Creating situations for students to professionally collaborate on complex patient cases can raise learners’ appreciation for why input from other disciplines is relevant and necessary (Ng et al., 2021; Roberts et al., 2018).

Their experiences at the clinic consolidated an appreciation that collaboration across professional boundaries can amplify any one profession’s capacity to care for patients with complex needs (Ng et al., 2021).

Learning and working together in situations where professionals are interdependent for effective problem-solving may increase respect (Friman et al., 2017; Smith et al., 2020), trust (Berger-Estilita et al., 2020a, b), valuing of collaboration, and result in greater appreciation for other professionals’ skills and contributions to patient care (Ng et al., 2021; Smith et al., 2020; Stephens & Ormandy, 2018). The experienced mutual interdependence promotes the development of a dual identity (Lockeman et al., 2017), that is, a profession-specific identity (Bloomfield et al., 2021; McGregor et al., 2018) and an interprofessional identity (Bloomfield et al., 2021; Lockeman et al., 2017; Matulewicz et al., 2020; Seaman et al., 2018). This is summarized by Lockeman et al., (2017, p. 33) as follows:

Practitioners develop an interprofessional professional identity as a collaborator that complements each individual’s profession-specific professional identity (…). Studies among practitioners have shown that interprofessional education helps to redefine professional identities consistent with the dual identity model (…).

Sharing responsibility for complex patient care may also reduce hierarchy by highlighting how different team members make valuable contributions for optimal patient care delivery (Berger-Estilita et al., 2020a). The collaborative experiences in IPE also results in transformative learning, that is, learning that allows previous beliefs and assumptions of the learner to be reshaped (Muzyk et al., 2019; Ng et al., 2021).

(…) our research showed that the clinical experience in an SRFC helped facilitate a shift in attitudes, knowledge, and comfort working with other healthcare professions.(Ng et al., 2021, p. 707)

*CMOC* 4: When IPE facilitators serve as role models to students by being experts in their field, reflective of their own practice, and cultivating feelings of equality (C), they contribute to positive attitude development (O) by displaying good behavior to emulate, eliciting positive affect in their learners, and positively shaping interprofessional relationships (M).

As learners are socialized into their roles and develop their professional and interprofessional identity (Friman et al., 2017; Lockeman et al., 2017) through social learning (Lockeman et al., 2017), IPE facilitators serve as role models for learners’ development.

Professionals form identities through a process of socialization (…). The process of socialization is influenced by multiple factors, including (…) role models and mentors, as well as formal teaching with faculty and self-assessment. (…) interactions and experiences can be developed by educators to help shape positive interprofessional relationships (…). (Lockeman et al., 2017, p. 33)

Having facilitators who are competent role models, who facilitate reflections (Berger-Estilita et al., 2020a), and cultivate feelings of equality between facilitator and learner (Stephens & Ormandy, 2018) may elicit positive affect during IPE and support positive attitude development by producing positive interactions and experiences and positively shaping interprofessional relationships (Lockeman et al., 2017). On the value of positive interactions, Stephens and Ormandy (2018) note that:

Students commented on the positive dynamics within the groups (…). Students across groups agreed that they felt a sense of equality between the group facilitators and the group members (…) (p. 352)

(…) the positive group dynamics had a significant effect on the student’s values, attitudes, and beliefs about each other (…). (p. 354)

A similar perspective on the value of facilitators as role models is described by Berger-Estilita et al., (2020a, p. 14):

(…) small-group reflections, facilitated by adequate role models, may allow students to remodel their own professional and personal attitude (…).

Conversely, less competent facilitators negatively influence learner perceptions, resulting in a decline in positive attitudes towards that facilitator’s profession. For example, when medical students were taught by non-physicians they perceived as less competent, they may express a lack of confidence in their IPE facilitator and their IPE instruction (Berger-Estilita et al., 2020a).

*CMOC* 5: When the IPE curriculum is perceived to be career relevant, boosts confidence, and increases learners’ comfort in working with other professions in patient care delivery (C), it leads to improved attitudes (O) because learners come to expect positive experiences from IP interactions and come to value these interactions more (M).

The attributes of IPE can also contribute to developing positive attitudes among learners, particularly when IPE is perceived as relevant to one’s career (Roberts et al., 2018). The design of the IPE curriculum can contribute to positive group dynamics (Stephens & Ormandy, 2018), leading to feelings of enjoyment and positive affect (Filies & Frantz, 2021; Mowat et al., 2017; Muzyk et al., 2019; Ng et al., 2021; Squires et al., 2021; Thompson et al., 2020). Eploring the experiences of nursing students in IPE, Thompson et al., (2020, p. 5) reported:

(…) they found the group to be very welcoming and respectful, and the session to be very relaxed. The results of this study also suggest that the nursing students became more confident as a result of the teaching; with some indicating that they would be happier to approach a doctor in the future (…).

IPE may contribute to positive affect by increasing professionals’ comfort in working with other professionals (Seaman et al., 2018), boosting confidence, and reducing feelings of inferiority (Thompson et al., 2020). Whereas having only limited interactions with other professionals may lead to feeling intimidated by them (Smith et al., 2020). The importance of curriculum relevance and the need to boost confidence in order to positively affect attitudes is summarized by Roberts et al., (2018, p. 39):

First, in order to maintain or increase positive attitudes towards IPE in introductory programs that span professions, the curriculum needs to be designed to demonstrate relevance to the future careers of participating students from all professions. Second, as IPE may be particularly challenging for students who do not have confidence in their abilities to communicate and work effectively in teams, educators may need to focus on building these skills to decrease negative attitudes.

Whereas high quality IPE may boost positive attitudes, poor quality IPE can lead to boredom, frustration, negative attitudes, and students finding IPE unnecessary (Berger-Estilita et al., 2020a). A further IPE characteristic that can contribute to more positive attitudes is when learners are motivated to improve their own attitudes, such as when they become IPE instructors in their organization, thereby becoming advocates for interprofessional collaboration (Yang et al., 2017, p. 9):

When trying to improve each health professional’s IPC attitude with limited resources, including the time needed to carry out the training, the number of faculty members needed to run the training and the facilities needed for the training, each newly-trained participant should act as a seed instructor within their team. In other words, successful training of seed instructors can result in profession-wide IPC promotion and attitude remodelling.

#### Post-Program

*CMOC* 6: When there is organizational support for IPC and healthcare team members participate in IPE on an ongoing basis (C), sustained positive attitudes towards IPC are more likely (O) because the attitudes and values expected in IPC are continually positively reinforced, and are eventually integrated into the learners’ personal value system.

When the goal of developing positive attitudes in learners is achieved, the question of long-term sustainability is important to address post-program. The literature evidence suggests that positive IPC attitudes can be temporary without ongoing organizational efforts to reinforce them (Berger-Estilita et al., 2020a; Mowat et al., 2017; Snyman & Donald, 2019). Negative organizational culture towards IPC can dampen positive IPC attitudes and be passed on to next generations of staff (Friman et al., 2017). As noted by Squires et al. (2021, p. 197):

It is also important to note that even if students are prepared to be collaborative when they graduate, if the organizational culture does not support IPC and operates under traditional hierarchical, non-team friendly models, then the sustainability of graduates implementing the lessons learned about IPC from these programs is threatened.

Maintaining positive attitudes is also supported by continued IPE attendance (Mowat et al., 2017), which is more likely to occur if IPE is experienced as positive (Squires et al., 2021), relevant (Roberts et al., 2018), comfortable and open for attendees from different disciplines (Matulewicz et al., 2020). Sustained attitude change is more likely when attitudes are internalized and correspond to learners’ own values (Stephens & Ormandy, 2018, p. 349):

The first two stages of development (compliance and identification) are types of conformity and can revert to previously held attitudes and values, as they are both extrinsically motivated and require constant reinforcement. However, the third-stage internalisation is when a student embraces the new values and they become part of their belief system (…).

### Answering the realist research question

Building upon CMOCs 1–6, we answer the realist question as follows: IPE appears to work by providing formal and informal space for learners to socialize, which fosters trust, respect, and liking among the learners because it allows them to get to know each other professionally and personally. IPE appears to work positively for all learners but will differentially affect learners in different fields of study because they are likely to have different backgrounds and different attitudes and values prior to beginning their studies. Medical students have been noted to be more difficult to positively influence, whereas other professions, notably nursing students, may more easily develop positive IPE and IPC attitudes. When learning conditions and IPE facilitators produce positive affect in their learners and when learners experience that they need each other to solve practice problems, they are more likely to develop positive attitudes towards each other and towards collaboration.

## Discussion

This study developed a realist theory of how IPE affects learner attitudes towards IPE, IPC, and members of other healthcare professions than one’s own. This was achieved by conducting a realist synthesis, which identified requisite contextual factors necessary to trigger mechanisms resulting in intended outcomes. The analysis was iterative and built a successively more complex theory of how IPE affects learner attitudes, helping to understand “what works, for whom, how, and in what circumstances” (Pawson et al., 2005, p. 32).

### Explaining our findings with middle-range theories

We located several MRTs associated with our CMOCs. We present these associations by phase of IPE (See Table 5). The following sections discuss key MRTs associated with CMOC mechanisms.

#### Pre-Program

CMOC 1 suggests that attitudes and values already present before beginning university studies may contribute to IPE having less impact on some students, while having more impact on others. This proposition is supported by Social Judgment Theory (Sherif & Hovland, 1961), which suggests that existing attitudes may serve as anchors against which new information is judged. Existing attitudes will impact the probability that new ideas presented in IPE will be accepted or rejected. Especially when an idea being judged is closely tied to a learner’s self-concept and values, the range of ideas that would be considered acceptable becomes narrower and the higher the chances are that a new idea will be rejected (Park et al., 2007).

There are several likely factors related to personal identity that explain why there might be already pre-existing attitudes formed before learners begin their healthcare studies. Some of these factors are presented in the PT figure (Fig. 2), such as gender, age, social background. For example, people develop a preference for specific work activities that are associated with their attitudes and values, and they prefer work environments congruent with their personality (Chen & Simpson, 2015; Holland, 1985). Some disciplines, such as medicine, may be best aligned with a personality that favors hierarchical differentiation. Social and cultural values can also influence individuals’ preference for careers based on status, prestige and social dominance orientation (Pratto et al., 1994; Sidanius & Pratto, 2001). Some research evidence suggests that preference for hierarchical differentiation among disciplines is inversely related to positive attitudes towards IPC (Ballard, 2016). This might explain why medical students who are aspiring to a high-status profession may have more negative attitudes towards interprofessional collaboration compared to students from other professions. In contrast, having a pro-social personality was found to influence job self-selection such that pro-social nurses tended to choose posts with more difficult working conditions (Lagarde & Blaauw, 2014).

#### Program

CMOC 2 proposes that when IPE provides time and facilities for formal and informal interactions, learners get to know each other professionally and personally, resulting in the intermediate factors mutual trust, respect and liking which are linked to positive attitudes towards IPC. An MRT that supports the importance of regular contact is Allport’s (1954) Contact Theory, also known as Intergroup Contact Theory. This theory suggests that, given the right conditions, contact between groups can affect a reduction in prejudice, conflict, and lead to an improvement in attitudes. Contact is more likely to lead to improved attitudes when there is equal status and respect among group members and when they work towards a common goal (Ballard, 2016; Bridges & Tomkowiak, 2010).

CMOC 3 suggests that when learners are put in settings where they need to work together to overcome problems, they develop an interprofessional identity and learn to respect and trust each other because they are able to observe how they can help each other in patient care and they come to realize that they all depend on each other. This proposition is given support by Social Identity Theory (Tajfel et al., 1979), which suggests that individuals construct their identity, at least in part, from their social group (Ballard, 2016). This can lead to in-group preference, wherein healthcare professionals favor members of their own profession over members of other professions (Biehle et al., 2019). However, through IPE, the development of a dual social identity is facilitated, which is composed of a professional identity and an interprofessional identity. When a social identity is developed as part of the interprofessional group, it is an example of decategorisation as part of the out-group and recategorisation as part of the in-group, as predicted by Social Identity Theory (Tajfel et al., 1979). These processes help in deemphasizing the salience of group distinction (Ballard, 2016). We believe this deemphasis of group distinction may be supported by learners experiencing their mutual interependence among each other to solve problems in everyday practice (Maddock et al., 2023). Deemphasis of group distinction and emphasis of joint group identity as an interprofessional team may also help learners to attribute positive characteristics to members of the other profession and to like them more, as they are now considered members of one’s own interprofessional group.

CMOC 4 recognizes the importance of IPE facilitator role modeling. As proposed by Social Learning Theory (Bandura & Walters, 1977), learning takes place through observing and modeling the attitudes and behaviors of others. Thus, when learners observe good role models who are reflective, cultivate feelings of equality, and contribute to positive interactions within the group, learners are likely to emulate them.

CMOC 5 acknowledges how well-designed IPE curricula must be perceived as relevant to learners’ careers as well as valuable and achievable. According to Expectancy-Value Theory (Wigfield & Eccles, 2000), when tasks such as IPE or IPC are deemed relevant and valuable to learners, and learners expect that they can achieve the objectives, they are more likely to engage in the activity and develop positive attitudes towards it. Indeed, Expectancy-Value Theory formed part of the theoretical foundation for the development of a questionnaire instrument measuring behavioral confidence to undertake interprofessional education activities (Blumenthal et al., 2022).

#### Post-Program

Dealing with the post-program mechanism of IPE, CMOC 6 proposes that when IPE receives organizational support and is attended on a continuing basis, team members are more apt to sustain their positive attitudes towards IPC. The Affective Domain Development Model developed by Epstein (1977) lends support to this proposition. The model developed with nursing students suggests a three-stage development process of values, attitudes, and behavior. In the first stage of development, learners merely assume or conform to the expected attitudes and behavior. However, in the last stage, the stage of internalization, the new attitudes and values are embraced for their intrinsic value because they have become part of the learners’ value system. This model was recently applied to another study on the development of attitudes among nurses, which used a validated assessment tool to track nurses’ attitude formation over time (Stephens & Ormandy, 2019).

The elaborated links between our developed CMOCs and MRTs provide additional theoretical validation for the underlying proposed mechanisms of IPE in producing positive attitude outcomes.

### Implications for practice

The developed CMOCs highlight the manifold social processes embedded in IPE teaching (Hean et al., 2018), which can be shaped by IPE curriculum developers and IPE facilitators. A key finding of our research indicates the importance of trust, respect, and mutual liking in developing positive attitudes. IPE that facilitates formal and informal interactions through group projects, joint clinical placements, and social events are likely to provide such suitable settings. Our finding that IPE needs to be perceived as relevant for one’s career suggests that the alignment of course content with real-world healthcare scenarios is particularly valuable for engendering positive attitudes. Creating IPE experiences where multiple professions have to collaborate to solve complex patient cases and where learners can experience the mutual dependence of the various professions may be especially valuable in emphasizing the relevance of IPC. IPE facilitators need to be mindful that they shape how a course is experienced by learners and how they themselves are perceived. If they are perceived as competent in their roles and are able to elicit positive group interactions and positive affect in their learners, it can contribute to learners having a more positive attitude towards IPE and towards their fellow learners. Finally, adopting a more theory-driven approach towards curriculum development and IPE facilitation may more likely yield positive attitude outcomes, as one can benefit from previous knowledge of what is likely to have the desired effect, under which conditions, and for which learners. Our CMOCs can provide guidance as to which contexts are conducive to triggering the mechanisms that may increase the effectiveness of IPE in influencing sustained positive attitudes.

### Strengths and limitations

This rapid realist review synthesizes 12 years of studies on IPE and positive attitude development. Applying realist synthesis, a key strength of this approach was its iterative analysis of data and validation by SMEs. The refined PT and the six CMOCs are testable explanations for what works, for whom, how and in what circumstances to influence positive attitude development towards IPE and IPC. The PT is a placeholder for contextual factors to consider in the progression from one professional identity to a profession-specific and interprofessional dual identity. The CMOCs are hypotheses that provide plausible explanations for what works in the real world. They also can be used for theory-building, considering the number of existing MRTs that may be associated with the mechanisms in our six CMOCs.

Because this was a rapid realist synthesis, we uncovered some important contextual factors, such as gender, age, social/cultural background, aspired profession and personality type. We have included them as factors to consider in the program theory (Fig. 2), but we were unable to delve into all related literature and develop CMOCs for this diversity of contextual factors. A further study limitation lies in the possibility of subjectivity in the interpretation of textual data, although the research team included IPE SMEs and realist methodologists. Finally, given the short period of time for this project, the PT and CMOCs may not capture all potential intermediate causal mechanisms. For example, there may be other mechanisms and theory associated with discipline-specific attitudes and the transition to a dual identity. We believe this paper provides further theoretical advancement of how IPE can result in sustained positive IPC attitudes.

## Conclusion

This realist synthesis sets the stage for appreciating contextual factors and associated mechanisms resulting in positive IPE and IPC attitudes. Key mechanisms of positive attitude development include getting to know the other learners professionally and personally, experiencing positive affect during IPE, and learners experiencing mutual dependence. IPE that facilitates formal and informal interactions, is led by competent facilitators, and is perceived as career-relevant may provide the conducive contexts to trigger such mechanisms.

## Supplementary Information

Below is the link to the electronic supplementary material.

Supplementary file1 (DOCX 18 KB)

Supplementary file2 (DOCX 61 KB)

## Data Availability

Data sets analysed in the current study are available from the corresponding author on reasonable request.
