# Supplementary material for: How does interprofessional education affect attitudes towards interprofessional collaboration? A rapid realist synthesis
Source: Adv Health Sci Educ Theory Pract. 2024 Sep 23;30(3):879–933. doi: 10.1007/s10459-024-10368-6 (PMC12119706; doi:10.1007/s10459-024-10368-6)
Supplement: Supplementary file 1 — Supplementary file1 (DOCX 18 KB) [file 10459_2024_10368_MOESM1_ESM.docx]

**Additional File 1: Full search strategy**

**PubMed**

(("Inter-professional education"[All Fields] OR "Interprofessional Education"[All Fields] OR "interprofessional training*"[All Fields] OR "inter professional training*"[All Fields] OR "multi-professional education"[All Fields] OR "multiprofessional education"[All Fields] OR "multi professional training*"[All Fields] OR "multiprofessional training*"[All Fields] OR "common learning"[All Fields] OR "shared learning"[All Fields] OR "interdisciplinary learning"[All Fields] OR "Interprofessional Education"[MeSH Terms]) AND (("attitude*"[All Fields] AND ("change*"[All Fields] OR "development*"[All Fields])) OR "Attitude of Health Personnel"[MeSH Terms] OR "interpersonal relations"[MeSH Terms]) AND ("healthcare provider*"[Title/Abstract] OR "health care provider*"[Title/Abstract] OR "healthcare worker*"[Title/Abstract] OR "health care worker*"[Title/Abstract] OR "Allied Health"[Title/Abstract] OR "health profession*"[Title/Abstract] OR "Health Occupations"[MeSH Terms] OR "students, health occupations"[MeSH Terms] OR "Health Personnel"[MeSH Terms] OR "Doctor"[Title/Abstract] OR "Physician"[Title/Abstract]) AND ((("interprofessional"[All Fields] OR "interprofessionalism"[All Fields] OR "interprofessionality"[All Fields] OR "interprofessionally"[All Fields] OR "interprofessionals"[All Fields]) AND ("collaborate"[All Fields] OR "collaborated"[All Fields] OR "collaborates"[All Fields] OR "collaborating"[All Fields] OR "collaboration"[All Fields] OR "collaborations"[All Fields] OR "collaborative"[All Fields] OR "collaborative s"[All Fields] OR "collaboratively"[All Fields] OR "collaboratives"[All Fields] OR "collaborator"[All Fields] OR "collaborators"[All Fields])) OR (("interprofessional"[All Fields] OR "interprofessionalism"[All Fields] OR "interprofessionality"[All Fields] OR "interprofessionally"[All Fields] OR "interprofessionals"[All Fields]) AND ("practicability"[All Fields] OR "practicable"[All Fields] OR "practical"[All Fields] OR "practicalities"[All Fields] OR "practicality"[All Fields] OR "practically"[All Fields] OR "practicals"[All Fields] OR "practice"[All Fields] OR "practice s"[All Fields] OR "practiced"[All Fields] OR "practices"[All Fields] OR "practicing"[All Fields])) OR (("interprofessional"[All Fields] OR "interprofessionalism"[All Fields] OR "interprofessionality"[All Fields] OR "interprofessionally"[All Fields] OR "interprofessionals"[All Fields]) AND ("team s"[All Fields] OR "teamed"[All Fields] OR "teaming"[All Fields] OR "teamness"[All Fields] OR "teams"[All Fields])) OR (("interdisciplinary studies"[MeSH Terms] OR ("interdisciplinary"[All Fields] AND "studies"[All Fields]) OR "interdisciplinary studies"[All Fields] OR "interdisciplinary"[All Fields]) AND ("teamwork"[All Fields] OR "teamworking"[All Fields])) OR ("cooperative behaviour"[All Fields] OR "cooperative behavior"[MeSH Terms] OR ("cooperative"[All Fields] AND "behavior"[All Fields]) OR "cooperative behavior"[All Fields]))) AND ((humans[Filter]) AND (english[Filter]) AND (2011:2022[pdat]))

**CINAHL**

**Concept 1: Interprofessional Education**

TI ( "Inter-professional education" OR "interprofessional education" OR "interprofessional training*" OR "inter-professional training*" OR "multi-professional education" OR "multiprofessional education" OR "multi-professional training*" OR "multiprofessional training*" OR "common learning" OR "shared learning" OR "interdisciplinary learning" ) OR AB ( "Inter-professional education" OR "interprofessional education" OR "interprofessional training*" OR "inter-professional training*" OR "multi-professional education" OR "multiprofessional education" OR "multi-professional training*" OR "multiprofessional training*" OR "common learning" OR "shared learning" OR "interdisciplinary learning" )

**Concept 2: Attitude**

TI (Attitude* AND (change* OR development*)) OR AB (Attitude* AND (change* OR development*))

**Concept 3: Healthcare professionals or students**

TI ("healthcare provider*" OR "health care provider*" OR "healthcare worker*" OR "health care worker*" OR "Allied Health Personnel*" OR "Allied Health" OR "Physician*" OR "Doctor*" OR Health Profession*)

**Concept 4: Interprofessional Collaboration**

TI (“Interprofessional collaboration” OR “Interprofessional practice*” OR “Interprofessional team*”

OR “interdisciplinary teamwork” OR “cooperative behavior”)

**PsycInfo**

**Concept 1: Interprofessional Education**

("Inter-professional education" or "interprofessional education" or "interprofessional training*" or "inter-professional training*" or "multi-professional education" or "multiprofessional education" or "multi-professional training*" or "multiprofessional training*" or "common learning" or "shared learning" or "interdisciplinary learning").ti OR ("Inter-professional education" or "interprofessional education" or "interprofessional training*" or "inter-professional training*" or "multi-professional education" or "multiprofessional education" or "multi-professional training*" or "multiprofessional training*" or "common learning" or "shared learning" or "interdisciplinary learning").ab

**Concept 2: Attitude**

(Attitude* AND (change* OR development*) OR value* OR perception*).ti OR (Attitude* AND (change* OR development*) OR value* OR perception*).ab

**Concept 3: Healthcare professionals or students**

("healthcare provider*" OR "health care provider*" OR "healthcare worker*" OR "health care worker*" OR "Allied Health Personnel*" OR "Allied Health" OR "Dentist*" OR "Emergency Medical Dispatcher*" OR "Paramedic*" OR "Nurse*" OR "Nursing Aide" OR "Occupational Therapist*" OR "Physical Therapist*" OR "Therapist*" OR "Physician*" OR "Doctor*" OR midwives OR midwife OR Health Profession*) OR (student* AND "healthcare worker*" OR "health care worker*" OR "Allied Health Personnel*" OR "Allied Health" OR "Dentist*" OR "Emergency Medical Dispatcher*" OR "Paramedic*" OR "Nurse*" OR "Nursing Aide" OR "Occupational Therapist*" OR "Physical Therapist*" OR "Therapist*" OR "Physician*" OR "Doctor*" OR midwives OR midwife OR Health Profession*).ti OR ("healthcare provider*" OR "health care provider*" OR "healthcare worker*" OR "health care worker*" OR "Allied Health Personnel*" OR "Allied Health" OR "Dentist*" OR "Emergency Medical Dispatcher*" OR "Paramedic*" OR "Nurse*" OR "Nursing Aide" OR "Occupational Therapist*" OR "Physical Therapist*" OR "Therapist*" OR "Physician*" OR "Doctor*" OR midwives OR midwife OR Health Profession*) OR (student* AND "healthcare worker*" OR "health care worker*" OR "Allied Health Personnel*" OR "Allied Health" OR "Dentist*" OR "Emergency Medical Dispatcher*" OR "Paramedic*" OR "Nurse*" OR "Nursing Aide" OR "Occupational Therapist*" OR "Physical Therapist*" OR "Therapist*" OR "Physician*" OR "Doctor*" OR midwives OR midwife OR Health Profession*).ab

**Concept 4: Interprofessional Collaboration**

("interprofessional collaboration" or "interprofessional practice" or "interprofessional team" or "interdisciplinary teamwork" or "cooperative behavior").ti,ab.
