# Supplementary material for: How does interprofessional education affect attitudes towards interprofessional collaboration? A rapid realist synthesis
Source: Adv Health Sci Educ Theory Pract. 2024 Sep 23;30(3):879–933. doi: 10.1007/s10459-024-10368-6 (PMC12119706; doi:10.1007/s10459-024-10368-6)
Supplement: Supplementary file 2 — Supplementary file2 (DOCX 61 KB) [file 10459_2024_10368_MOESM2_ESM.docx]

**Quotes from reviewed studies**

| **Quote** | **Study** | **Bucket** | **Quote** | **Page** |
| --- | --- | --- | --- | --- |
| 1 | Berger-Estilita 2020a | IPE characteristics | (...) IPE should start as early as the first year of studies. They mentioned several advantages for early IPE introduction which included (1) easier implementation (as students would have similar backgrounds) and (2) the encouragement of early interaction, shared learning and networking, which would contribute to the building of mutual respect from an early stage. Students suggested starting with basic science and other overlapping top- ics, which could then evolve to clinical interactions later in the curriculum. | 12 |
| 2 | Berger-Estilita 2020a | IPE characteristics | One third of students mentioned the importance of the early introduction of IPE in the curriculum, as it facilitated an early interaction and network, contributing to mutual respect and reducing stereotypes. Thus, students can join an interprofessional team without bringing a well-developed “doctor professional identity” [34]. Social Identity Theory [36] supports this: stronger definitions of individual professional roles may lead to intergroup discrimination. Introducing IPE early in the curriculum is likely to have an impact on students’ ability to assume their given roles and responsibilities, which is a basic principle of professionalism [37]. Finally, having to learn interprofessional teamwork skills in the workplace in addition to clinical responsibilities and patient care, may increase extraneous cognitive load [38, 39]. Learning these skills may be better served within basic sciences courses, as they provide a more favour- able framework for the initiation of IPE [40] Early introduction of IPE would also tackle lower levels of prejudice, promoting more positive attitudes [41]. | 14 |
| 3 | Berger-Estilita 2020a | IPE Characteristics | Students favored regular IPE interventions, with course repetitions. Participants did not agree on an adequate frequency: while some wished for IPE to occur on a weekly, fortnightly or monthly basis, others preferred only once or twice every semester. Some students were con- cerned about the time it would take to prepare for weekly IPE (e.g., communication) trainings. Regarding the topic of the IPE intervention, students chose basic science topics for pre-clinical years (including anatomy, biology and patient confidentiality). | 13 |
| 4 | Berger-Estilita 2020a | - IPE characteristics - IPE learner development/transformation | Introducing IPE early in the curriculum is likely to have an impact on students’ ability to assume their given roles and responsibilities, which is a basic principle of professionalism. | 14 |
| 5 | Berger-Estilita 2020a | - IPE characteristics - IPE learner development/transformation | One third of students mentioned the importance of the early introduction of IPE in the curriculum, as it facilitated an early interaction and network, contributing to mutual respect and reducing stereotypes. (...) Early introduction of IPE would also tackle lower levels of prejudice, promoting more positive attitudes. | 14 |
| 6 | Berger-Estilita 2020a | IPE learner development/transformation | All participants mentioned that it was a positive experience and that they profited from the course. Main positive aspects mentioned included: (1) the teaching and then the practice with a skilled nursing student; (2) the relaxed, informal interaction; and (3) the exchange of information and guidance from the nursing students, with tips from daily practice. | 7 |
| 7 | Berger-Estilita 2020a | IPE learner development/transformation | The social component of IPE was mentioned as a goal and as an advantage. Students considered the networking beneficial, and by engaging on interprofessional relationships on a personal level, they could learn about each other’s curricula in informal settings and even foster friendships. This is a point not frequently explored in the literature. The social aspect repeatedly mentioned in the interviews mirrors many of the components of Social Learning Theory [56]. Learning is also a social and relational process, frequently occurring around authentic and meaningful patient cases [45, 49]. Such findings show that “formal” or planned educa- tional IPE experiences also create “informal” opportunities to socialise and be acquainted on a personal level. These “informal arenas can, therefore, stimulate and set a solid basis for interprofessional collaboration” [54]. | 15 |
| 8 | Berger-Estilita 2020a | - IPE learner development/transformation - IPE characteristics | If the IPE experience is not perceived as good by all students, there is a risk that they will consider it unnecessary. The implementation of such activities may be challenging because the content, format and frequency rarely accommodate all students involved. There was a frequently mentioned fear that students would not benefit from the topics due to their diverse backgrounds or varying levels of knowledge on a given subject. Medical students were con- cerned that topics would be approached too superficially. This could lead to boredom and frustration or create a feeling of unworthiness. | 11 |
| 9 | Berger-Estilita 2020a | - IPE learner development/transformation - IPE teaching/learning approaches | Students were regardful that finding commonalities in different healthcare professions intensifies social relations both inside and outside the workplace, leading to a social benefit. | 11 |
| 10 | Berger-Estilita 2020a | - IPE learner development/transformation - IPE teaching/learning approaches | Students would rather have IPE in smaller groups (4–6 participants, mixed ratio 1:1 or 1:2) to allow for a better interpersonal experience and communication. As for the preferred duration, they felt these should be course blocks of approximately 1–4 hours, entailing a full morn- ing or afternoon. **IP courses should have an optional character**. | 13 |
| 11 | Berger-Estilita 2020a | - IPE learner development/transformation - Learner background, characteristics | Factors contributing to this decline in interprofessional attitudes include being more experienced in the healthcare field [32], having previous interprofessional contact [42], having had less positive experiences in IPE [31, 34, 43] and having parents working in healthcare [44]. Although specifically targeted for the Bernese sample, none of these factors showed a significant association with the decline in attitudes. | 14 |
| 12 | Berger-Estilita 2020a | - IPE learner development/transformation - Learner background, characteristics | Students had an outspoken fear of loss of medical identity and some showed no positive attitudes towards interprofessionality. Others, despite being at the beginning of their professional career, showed a stereotypical view and regarded interaction between health professions as difficult, which is similar to previous findings [49–52]. | 14 |
| 13 | Berger-Estilita 2020a | - IPE learner development/transformation - Teaching environment: facilitators | The decline in students’ attitudes towards IPE observed in the quantitative analysis, coupled with 30% of the participants mentioning clear disadvantages of early IPE implementation is worrisome. This is of concern because good relationships with colleagues and patients–likely fostered by IPE–increase patient satisfaction, promote treatment compliance and protect against malpractice claims [46]. Hudson et al. [34] suggested this may be due to the nature of the intervention and how negatively students experienced it. Being taught by non-doctors also reduces medical students ́ motivation to participate in IPE interventions [34]. The arguments above, coupled with an underdeveloped professional identity, may have been the reason for the decline. On-going team training may tackle this, as it has been shown to be central in the sustainability of a shared understanding of professional roles [47, 48]. | 14 |
| 14 | Berger-Estilita 2020a | IPE teaching/learning approaches | Courses should be practical (tutorials, case studies, clinical skills trainings, problem-based learning groups, case- based learning) and lectures should be avoided. Other options mentioned included seminars or course days about topics which are relevant to more than one profession or the use of simu- lation for soft skill and clinical skill training. Some students recommended that such courses should occur during clinical rotations and include other healthcare students. The IP groups should, when possible, be maintained throughout the year to allow for a deeper social interaction. | 12 |
| 15 | Berger-Estilita 2020a | IPE teaching/learning approaches | In the present study, students favoured regular IPE to maintain interprofessional proficiency. Both findings reinforce the need to offer health care professional students enough opportunities to interact and learn together from the first year of studies and throughout their careers. | 14 |
| 16 | Berger-Estilita 2020a | IPE teaching/learning approaches | It was also noted that if groups were not deliberately mixed, students from the same profession tended to group together and quality learning was impacted. A medical student who had a nursing background added: | 9 |
| 17 | Berger-Estilita 2020a | - Learner attitudes, perspectives, behaviors - IPE teaching/learning approaches | Reasons opposed to an early IPE introduction included students being overwhelmed by an overloaded, integrative year; the role of “doctor” not being yet clearly defined and prejudices against other health care professions existing before medical school. On the other hand, eleven students pointed out that the IPE introduction should occur just before or during clinical years (from the third year onwards). For them, it meant a better integration of the IPE content with clinical practice, the previous acquisition of basic clinical knowledge which would facilitate the focus on the IP component, and the broader diversity of activities that could be offered. One student was concerned that such an approach would be too late to prevent the development of prejudices. Five students mentioned it was important to have IPE on a frequent, recurrent basis. | 12 |
| 18 | Berger-Estilita 2020a | - Learner attitudes, perspectives, behaviors - Learner background, characteristics | (…) most students realised that nursing students already had the given competency and were bored/frustrated during the workshop. Some medical students observed other peers having discriminating attitudes towards nursing students. Most were unhappy to be in a workshop where they knew less than their nursing counterparts and could not contribute to any exchange in knowledge. | 7-8 |
| 19 | Berger-Estilita 2020a | - Learner attitudes, perspectives, behaviors - Teaching environment: barriers | Unfortunately, stereotypes formed by professional interaction and societal views on professional roles are not easily modified by educational interactions alone [54]. The introduction of small-group reflections, facilitated by adequate role models, may allow students to remodel their own professional and personal attitude towards patients, to express their moral judgements from their observations of other healthcare professionals’ interactions and to share these experiences within a safe learning environment [48]. | 14 |
| 20 | Berger-Estilita 2020a | Learner background, characteristics | Females had significantly more positive attitudes towards interprofessionality in the overall G-IPAS and for the subscales of “teamwork, roles and responsibilities” and “patient-centeredness”. Selected studies from Sweden [28, 29], using either the RIPLS or the Jefferson Scale also showed more positive attitudes towards teamwork in females. Others [30] reported a significant effect of gender in the IEPS empathy subscale. No other studies seem to report such a gender effect. | 13 |
| 21 | Berger-Estilita 2020a | - Learner background, characteristics - Teaching environment: barriers | Issues regarding the competition with the current medical curriculum, the risk of unbalanced learning and other dangers were explored. Students feel they already have an overloaded sched- ule, so additional IPE interventions could be difficult to implement. They were uncomfortable with being taught by non-doctors because they feared other health care professionals would not be aware of their training or be knowledgeable about their curriculum. The lack of assess- ment of such activities labels IPE interventions as secondary, superfluous or less relevant. There was an outspoken fear of loss of medical identity, loss of medical specialization (because knowledge is shared), and fear of being less thorough in their own medical curriculum. | 11 |
| 22 | Berger-Estilita 2020a | Teaching environment: barriers | Most students hinted that the course was not well structured and that students did not mix, so the experience was not really IP. The reason for it being interprofessional was the common topic rather than the interaction between groups. | 9 |
| 23 | Berger-Estilita 2020a | - Values towards IPE - IPE teaching/learning approaches | Such interventions allow for exchange of knowledge or skills and sharing of different experiences, which improves understanding and communication between groups, and builds trust. | 7 |
| 24 | Berger-Estilita 2020a | - Values towards IPE - IPE teaching/learning approaches | All of these observations should be considered in order to offer more authentic interdisciplinary experiences, with the healthcare team and the patient engaging in interprofessional problem-solving activities. Such significant learning interactions have a clear impact on how medical students internalise and approach patient-centeredness [57]. | 15 |
| 25 | Berger-Estilita 2020b | - IPE characteristics - IPE learner development/transformation - Learner attitudes, perspectives, behaviors | The optimal timing to introduce IPE is still subject to debate [10]. In clinical years it may seem reasonable, as it contributes to optimal development of students’ professional identities and gives them experience in working collaboratively with students in different health professions [11]. However, the introduction of IPE so late in the medical curriculum may be complicated by the students’ focus on profession-specific clinical practice [10]. On the other hand, introducing IPE early in pre-registration healthcare courses may be useful in breaking down negative attitudes and avoiding stereotypes [58–60]. | 13 |
| 26 | Berger-Estilita 2020b | - Teaching environment: barriers/facilitators - Learner background, characteristics - Learner attitudes, perspectives, behaviors | The results from this review and from individual studies should be interpreted with caution: students’ educational backgrounds, as well as attitudes, expectations and stereotypes, may vary considerably between institutions and countries and may influence how the IPE interventions are experienced. | 14 |
| 27 | Biehle et al. 2019 | - IPE teaching/learning approaches - Learner attitudes, perspectives, behaviors | Interestingly, for physicians, who conversely were highly respected and closely aligned with pharmacist rankings during the pre-survey, the APPE appeared to lessen the opinion of pharmacy students towards physicians. The reason for this finding is unclear, but perhaps in this case the exposure served to “humanize” this traditionally highly respected profession that many students may have placed on a pedestal. | 421 |
| 28 | Biehle et al. 2019 | Values towards IPE | Despite the limitations, these results showed that the internal medicine APPE experience was able to change the perceptions of pharmacy students towards most other healthcare professionals assessed. For the non-physician professions, the APPE experience resulted in pharmacy student perceptions evolving to reflect more general equality in rankings across the various SSRQ categories. | 421 |
| 29 | Bloomfield et al. 2021 | IPE learner development/transformation | Findings revealed that most students rated the workshop positively and reported that it had changed their views of other health professionals. Many commented on their enjoyment meeting and interacting with students studying health degrees different from their own, and how this had benefitted them from both a social and educational perspective. | 6 |
| 30 | Filies and Frantz 2021 | IPE learner development/transformation  IPE teaching/learning approaches | Findings from the current study showed that the students valued collaborative learning with other healthcare professional students, as well as sharing experiences with them. Numerous studies have observed that health science students are inclined to report positive attitudes towards interprofessional education in general (Pollard et al., 2004; Tunstall-Pedoe et al., 2003). | 3 |
| 31 | Friman et al. 2017 | IPE learner development/transformation | In our study, nursing students experienced the physician’s uneasiness and lack of interest and knowledge in direct contact with wounds. It is also interesting that the medical students discussed the fear they had of appearing knowledgeable regarding wound care. This fear could be an obstacle to future interprofessional collaboration. The future physician might seek either more collaboration to learn more or less collaboration to hide gaps in knowledge. | 625 |
| 32 | Friman et al. 2017 | IPE learner development/transformation | As learning is also a relational and social process (Egan & Jaye, 2009), joint processing around authentic and relevant patient cases provides opportunities for students to experience the interplay of roles in wound treatment. As Khalili et al. (2013) concluded, IPE may increase the awareness of students’ own role constraints and opportunities to know and respect other profession’s responsibilities and competence. The literature advocates educational programmes early in the medical and nursing curricula to enhance a basis for effective patient care (Hall, 2005; Khalili et al., 2013). | 624 |
| 33 | Friman et al. 2017 | IPE learner development/transformation | This study explored nursing and medical students’ attitudes and perceptions towards each other’s role and interprofessional collaboration in wound care. The IPE activity became an arena to highlight roles and stereotypes in relation to wound care. Participants benefitted from gaining insight into each other’s knowledge and skills by using a shared language and getting a richer picture of a patient case by experiencing each other’s knowledge area, thereby becoming more comfortable in their future professional roles. | 624 |
| 34 | Friman et al. 2017 | Learner attitudes, perspectives, behaviors | Attitudes can be both positive and negative and concern both one’s own profession and others. Attitude has been suggested to be important in influencing interprofessional collaboration (Delunas & Rouse, 2014; Hansson et al., 2010a). Our data from the Jefferson scale showed no attitude change after the IPE activity in either student group. This could partly pertain to attitudes being formed slowly over time. Thus, a single IPE activity may be insufficient to demonstrate change on an attitude scale. Stereotypes formed by professional socialisation processes and expectations on professional roles in society are not easily changed with educational interventions (Reeves, 2000). The lack of changes could also relate to the fact that scores were already very high in the first measure in the comparison (i.e. a ceiling effect). | 624-625 |
| 35 | Friman et al. 2017 | Learner attitudes, perspectives, behaviors | Recent studies still report dysfunctional interactions between different professions (Hansson, Arvemo, Marklund, Gedda, & Mattsson, 2010a). This could be due to organisational deficiencies, but it could also relate to separate professional cultures nurturing negative attitudes towards other professions (Hall, 2005; Hansson et al., 2010a). Attitudes have been proposed as a key aspect in interprofessional collaboration, and these are already partly formed during education (Khalili, Orchard, Laschinger, & Farah, 2013). | 620 |
| 36 | Friman et al. 2017 | - Learner attitudes, perspectives, behaviors - Teaching environment: barriers | Care culture and traditions were perceived by medical students as controlling factors of hierarchy and easily transferred to the next generation. They discussed the abuse of power and how they had to be careful not to insult nurses during their clinical training. They highlighted their training to be leaders and that they were expected to give orders. The general view was that a certain hierarchical structure would always dom- inate because someone had to be ultimately responsible. | 624 |
| 37 | Friman et al. 2017 | Learner background, characteristics | Professional identities comprise values and perceptions of roles (Hall, 2005; Khalili et al., 2013). Future healthcare professionals are socialised into their roles in interaction with teachers, professionals, and patients in clinical practice. These processes often occur separately for each profession and are kept apart in a hierarchical healthcare system whereby professional groups continue to work within their own silos keeping experiences, values, and language separated (Abbot, 1988; Hall, 2005; Khalili et al., 2013). One way to bring these different cultures together and enhance collaborative approaches in patient care could be interprofessional education (IPE) in which students learn about other professions’ competences in addition to increasing their knowl- edge on a specific topic (Hammick, Freeth, Koppel, Reeves, & Barr, 2007). IPE increases the awareness of professional stereotypes and roles, thereby developing the ability to better communicate and share knowledge and skills (Ateah et al., 2011; Carpenter, 1995; Khalili et al., 2013). Furthermore, students with IPE experiences are more prepared to work together with other professions (Lumague et al., 2006; Wilhelmsson, Ponzer, Dahlgren, Timpka, & Faresjö, 2011). | 620 |
| 38 | Friman et al. 2017 | - IPE characteristics | Also medical students stressed the need for collaboration and linked it to the complete picture of the patient. They believed that everyone’s skills would benefit by helping each other. They expressed the wish to learn more about different professional roles during education: | 623 |
| 39 | Friman et al. 2017 | - Learner background, characteristics - Teaching environment: barriers | In line with other studies (e.g. Delunas & Rouse, 2014; Hood et al., 2014; Wilhelmsson et al., 2011), the nursing students in this study found more positive attitudes towards collaboration. Differences between professional nursing and medical cultures have been reported, whereby nurses already from the start are directed towards working in teams whereas physicians learn more independently in a competitive climate (Baker, Egan- Lee, Martimianakis & Reeves, 2011; Hall, 2005; Pecukonis, Doile & Bliss, 2008). This may explain why nursing students seemed to welcome teamwork and collaboration more than medical students. | 624 |
| 40 | Friman et al. 2017 | - Teaching environment: barriers - Learner background, characteristics | Students’ discussion about professional roles and collaboration revealed perceptions of various structures in practice that may hinder future collaboration. They generally felt that old patterns, such as authoritarian attitudes and hierarchies, were obstacles to patient care.   Nursing students linked authoritarian attitudes to the gender perspective. They experienced the older physicians, especially male physicians, as authoritarian, but stressed at the same time that their own expectations could possibly reinforce their approach. They expected that younger physicians would listen more to them than older ones. A general view of nursing students was that authoritarian attitudes are connected to the professional role. They discussed an ordergiving attitude of physicians, with an example of giving prescriptions to nurses in passing. These incidents were perceived as disrespectful causing stress, for example, in discussions during the patient rounds. They emphasised the independence of the nursing profession as it is taught, but did not experience this in practice. (...)  Even medical students discussed the authoritarian attitude from the gender perspective, but they linked it more to hierarchy and status. They described the difference between older and younger physicians where the elderly with their hierarchical status saw the nursing role as merely the performer of the physician’s prescriptions. | 623-624 |
| 41 | Friman et al. 2017 | Teaching environment: facilitators | The formal education arrangements also created informal spaces and opportunities to socialise and get to know each other on a personal level. | 624 |
| 42 | Fusco and Foltz-Ramos (2018) | Learner attitudes, perspectives, behaviors | In both cohorts, the pre-survey scores on the SPICE-R instrument were high (agree and strongly agree) for all items. Prior to the experience, students had a fair degree of awareness of the importance and value of interprofessional health care teams. This is an interesting finding in that IPE has just recently gained momentum on our campus and there have been very few deliberately scheduled IPE activities to date. Other studies have identified positive attitudes about interprofessional learning from all disciplines regardless of prior interprofessional learning experiences (Hood et al., 2014). These high baseline scores on the SPICE-R instrument may be a reflection of the increased emphasis being placed on team-based care in professional practice and the exposure students are getting to this concept as part of their introductory clinical rotations. Still, after the SBL experience students’ attitudes positively changed, supporting the importance and value of IPE experiences within the curriculum. | 650-651 |
| 43 | Fusco and Foltz-Ramos (2018) | IPE characteristics | Early implementation of IPE is important and may prevent the development of negative interprofessional attitudes (Hood et al., 2014). | 648 |
| 44 | Fusco and Foltz-Ramos (2018) | - IPE learner development/transformation - IPE characteristics | While the debriefing sessions were not recorded or analyzed it was noted that many students appreciated the opportunity to get to know other healthcare professional students and understand more of what their roles and responsibilities were. As this is the first interprofessional activity between pharmacy and nursing students, these findings are not unexpected. Pharmacy students found the SBL experience more realistic” when they compared them to other high-fidelity SBL experiences that they participated in alone. Given the nature of the simulation (e.g. inpatient acute care) there is a high likelihood that in practice pharmacists would be working closely with other healthcare professionals and so it may be true that these interprofessional simulations closer mirrored clinical practice than uni-professional simulations. | 651 |
| 45 | King and Violato (2021) | - Learner attitudes, perspectives, behaviors - Learner background, characteristics | The case of the outliers in the RM and M&D faculties is interesting as there may be some aspect of the participants’experience that caused them to have more negative interprofessional attitudes. Previous healthcare and/or IPE experience and maturity may be two possible reasons contributing to these results. RM is a masters level entry program, therefore these students have an undergraduate degree at entry into the program. The majority of the M&D students typically have an undergraduate degree, with some having a previous health professional designation. The other health science faculties do not require an undergraduate degree for entry into their programs. However, results in the literature about the impact of maturation and prior experience on changes in attitudes across health professions (Renschler et al., 2016) are varied. A review by Olson and Bialocerkowski (2014) reported that the capacity for attitude improvement may be greater among more mature-experienced students. Anderson and Thorpe (2008) found maturity to be a factor influencing engagement with interprofessional activities. Based on the current study and diverse results in the literature, further exploration about the implications of maturation on curriculum is required. | 129 |
| 46 | King and Violato (2021) | - IPE learner development/transformation - Learner attitudes, perspectives, behaviors | For example, attitude toward IPE has been reported lower as students approach graduation than it was at the start of a professional program (Coster et al., 2008) and may be a result of increased knowledge and realization of a lack of ability. The influence of the Dunning & Kruger effect has been demonstrated for other self-report IPC measures (Violato & King, 2019). | 128 |
| 47 | Lockeman et al. (2017) | Learner attitudes, perspectives, behaviors | As we hypothesized, an increase in SPICE-R2 ratings from pretest to posttest demonstrated that the series of IPSE experiences enhanced interprofessional learning among all students regardless of profession. This change is not surprising given previous research showing that IPSE is well-received and improves attitudinal outcomes towards interprofessional practice (Palaganas et al., 2016). More notable is how the IPSE series appears to have influenced the perception of stereotypes of each profession. Both our study and two other studies (Ateah et al., 2011; Liaw et al., 2014a) demonstrated the capacity of interprofessional education to impact professional stereotypes. | 35 |
| 48 | Lockeman et al. (2017) | - Learner background, characteristics - IPE learner development/transformation | Interprofessional education is a promising approach for achieving this aim (Greiner and Knebel, 2003). In contrast to the traditional model for professional formation which relies on role modeling by more experienced practitioners (Bleakley and Bligh, 2008), interprofessional education seeks to create a dual identity. Practitioners develop an interprofessional professional identity as a collaborator that complements each individual's profession-specific professional identity (Khalili et al., 2013). This theoretical goal for interprofessional education is supported by evidence. For example, Crawford et al. (2016) demonstrated that students from other professions perceive the nursing profession differently because of interprofessional education. Studies among practitioners have shown that interprofessional education helps to redefine professional identities consistent with the dual identity model (Ateah et al., 2011; Hood et al., 2014; Langendyk et al., 2015; Meyer et al., 2015). | 33 |
| 49 | Lockeman et al. (2017) | - Learner background, characteristics - IPE learner development/transformation | The aim of our study was to explore whether a series of IPSE experiences promoted changes in attitudes and stereotypes among nursing and medical students. We framed this study within the concept of social learning theory (Bandura, 1977) whereby senior students would have developed professional stereotypes from previous experiences in both the curriculum and through their extracurricular activities. We hypothesized that an extended series of IPSE experiences with a longitudinal team that spanned several sessions and focused on collaboration around acutely ill patients would challenge professional stereotypes and create a stronger interprofessional professional identity. | 33 |
| 50 | Lockeman et al. (2017) | Learner background, characteristics  Learner attitudes, perspectives, behaviors  IPE learner development/transformation | Improving collaboration among health professions is vital for safe, high quality care (Greiner and Knebel, 2003). However, as learners become practitioners, they are acculturated into a professional identity that often creates barriers to collaboration (Hall, 2005). Part of an individual's professional identity is defined by how the learner views other professions, and the stereotypes that develop during professional identity formation can affect interprofessional collaboration in clinical settings (Carpenter, 1995b). Professional identity in the form of professional affiliation can conflict with interprofessional collaboration among members of a team (Kvarnström, 2008). For example, the most common professional stereotype—nurses serving doctors in an unequal power dynamic—has been shown to have a detrimental effect on patient safety (Leonard et al., 2004). Improving how professionals perceive other professions is necessary to enhance collaboration and improve healthcare delivery. Professionals form identities through a process of socialization; as existing personal identities develop through communities of practice, personal and professional identities are shaped (Cruess et al., 2015). Social learning theory (Bandura, 1977) suggests that identity acquisition stems from these learning processes. The process of socialization is influenced by multiple factors, including the learning environment, peer and personal relationships, clinical and non-clinical experiences, role models and mentors, as well as formal teaching with faculty and self-assessment. Symbols and rituals, along with features of the hidden curriculum such as attitudes and treatment by patients, peers, health care professionals, and the public, also socialize professionals in identity formation. While the strength of influences for identity formation may vary among professionals, interactions and experiences can be developed by educators to help shape positive interprofessional relationships (Cruess et al., 2015). | 33 |
| 51 | Lockeman et al. (2017) | Learner background, characteristics  Teaching environment: barriers/facilitators | Perhaps, most noteworthy is the heterogeneity of impact of our intervention. When we compared nursing students and medical students on each measure, nursing and medical students showed similar increases in positive stereotypes of nurses, but the same was not true of stereotypes about doctors. Nursing students had a significant increase in positive perceptions of physicians while medical students had little change. These differences by profession have several possible causes worth further investigation.  Social learning theory (Bandura, 1977) posits these stereotypes likely stem from influences in the clinical learning environment. Role models, for example, are one major factor that shapes professional identity (Bleakley and Bligh, 2008). In this regard, the differences between professions may be a result of differences in response to the simulations, differences in preceding learning environments, or a combination of these and other factors. One possibility is these simulation experiences may have inspired both professions to view nursing more positively than prior clinical experiences but only nursing students to view physicians more positively than these students' preceding clinical experiences. Alternatively, medical students may have entered the simulation sessions with a perspective on physicians from preceding learning experiences which was supported but not changed for the better or worse by the activity. If true, these results demonstrate shortcomings of the current clinical learning environment for how nurses view both professions and physicians view nurses. In addition, IPSE appears to address these shortcomings, at least short-term. | 35 |
| 52 | Matulewicz et al. (2020) | Learner background, characteristics | Professional differences may also be important. We identified differences in attitudes across professions in quantitative data. In our sample, pharmacy students exhibited attitudinal scores in the middle of the range of the pretest measure for all students enrolled in the course. Their posttest scores, however, were well above those of students in the other disciplines and represented positive change significantly greater than that of students from dentistry and occupational therapy. | 600 |
| 53 | Matulewicz et al. (2020) | Learner background, characteristics  IPE characteristics | Two classroom-based IPE experiences appeared to affect perceived learning for health professions students and have a significant impact on attitudinal changes for pharmacy students. As we seek to define how IPE affects interprofessional practice and health outcomes, these findings help identify the role of early IPE on professional identity, interprofessional socialization, and, perhaps, intentionality regarding future interprofessional practice. | 601 |
| 54 | McGregor et al. 2018 | IPE learner development/transformation  IPE characteristics | IPE can benefit early learners in discovering their professional identities as a healthcare worker and member of a multidisciplinary team. | 12 |
| 55 | Mowat et al. 2017 | Learner attitudes, perspectives, behaviors  IPE characteristics  IPE learner development/transformation | The format of the event—blending lectures with case study discussions—seemed to be effective at creating a positive attitude towards IPE. Although this effect was not sustained six months later, attitudes did significantly improve immediately following the event. This positivity was also observed in participant interviews, in which participants reported feeling inspired to increase collaboration. | 1427 |
| 56 | Muzyk et al. 2019 | Learner attitudes, perspectives, behaviors | Following discussions on important topics such as stigma, personal bias, benefits of treatment interventions, and involving patients in their care decisions, students demonstrated positive attitudinal changes toward patients with SUDs. | 1797 |
| 57 | Muzyk et al. 2019 | IPE learner development/transformation  IPE teaching/learning approaches | We created a learning environment where students shared their learning, experiences, beliefs, and personal stories. Students’ growth, both as individuals and as a group, is supported by our findings concerning attitudinal changes toward interprofessionalism. We included students and faculty from different health professions who shared their roles and their perspectives about patient care in classroom discussions. Faculty involved all student voices in discussions and modeled collaborative approaches to patient care. We attribute the significant improvement in students’ attitudes toward interprofessionalism in all domains of assessment (unlike our previous course in which significant improvement was more limited,24 as noted above) to these deliberate course enhancements. | 1797-1798 |
| 58 | Ng et al. 2021 | IPE characteristics | Participants also reported that one of the main reasons for working at [student-run free clinic] was to collaborate and learn about other professions in an authentic clinical setting that carried responsibility, which they perceived was lacking in the formal curriculum. | 704 |
| 59 | Ng et al. 2021 | IPE learner development/transformation | Their experiences at the clinic consolidated an appreciation that collaboration across professional boundaries can amplify any one profession’s capacity to care for patients with complex needs. | 704 |
| 60 | Ng et al. 2021 | IPE learner development/transformation | We found that an interprofessional [student-run free clinic] experience with a reflective focus group component led to transformative learning with shifts toward self-awareness, systems thinking, team learning and care, individualized care, and interprofessional care. | 708 |
| 61 | Ng et al. 2021 | IPE learner development/transformation  IPE characteristics | Similarly, our research showed that the clinical experience in an [student-run free clinic] helped facilitate a shift in attitudes, knowledge, and comfort working with other healthcare professions. Collaborating with other professions in an authentic clinical setting provided a more nuanced understanding of scopes of practice and the value of collaboration in providing comprehensive, client-centered care. | 707 |
| 62 | Roberts et al. 2018 | IPE learner development/transformation  IPE teaching/learning approaches | In summary, the findings of this research with a large cohort of first-year university students from 25 health science professions enrolled in a first-year interprofessional program highlight two fronts on which educators can focus to strengthen attitudes towards IPE. First, in order to maintain or increase positive attitudes towards IPE in introductory programs that span professions, the curriculum needs to be designed to demonstrate relevance to the future careers of participating students from all professions. Second, as IPE may be particularly challenging for students who do not have confidence in their abilities to communicate and work effectively in teams, educators may need to focus on building these skills to decrease negative attitudes. | 39 |
| 63 | Roberts et al. 2018 | Learner background, characteristics | The results indicate that positive IPE attitudes were more strongly endorsed than negative IPE attitudes, and that there is already a strong sense of professional identity in these first-year undergraduate students. | 36 |
| 64 | Roberts et al. 2018 | IPE characteristics | The importance of perceived relevance to adult learners is highlighted in Knowles (1970) “readiness to learn” andragogy principle, where “People become ready to learn something when they experience a need to learn it in order to cope more satisfyingly with real-life tasks or problems” (p. 44). Perceived relevance has been suggested as a necessary condition for successful IPE (Parsell & Bligh, 1999). Qualitative and mixed methods stu- dies have suggested that some students do not perceive IPE activities as relevant to their future careers (Roberts & Forman, 2014; Rosenfield, Oandasan, & Reeves, 2011) or only appreciate the relevance once they begin professional practice (Pollard, Rickaby, & Miers, 2008). | 34 |
| 65 | Seaman et al. 2018 | IPE characteristics | Both student groups suggested the most appropriate time for interprofessional learning is earlier. The ISVS score was high both before and after placement, indicating they thought interprofessional skills were important, but not at the expense of developing profession-specific skills. This suggests the experience should be at the start of a degree not at the end. | 3128 |
| 66 | Seaman et al. 2018 | IPE learner development/transformation | A clinical placement in an ambulatory setting for nursing and medical students supported by a nurse educator and teaching registrar resulted in an increase in self-perceived ability to work with others and comfort in and valuing working with others. | 3128-3129 |
| 67 | Seaman et al. 2018 | IPE learner development/transformation  IPE teaching/learning approaches | The findings from this study suggest that students valued the ambulatory care placement and that their attitudes, awareness and behaviours in working with others from different disciplines positively changed as a result of this 2-week placement. The gains appeared greater for nursing students, and although there was overlap in the benefits noted by both medical and nursing students, there were differences in the challenges they identified. | 3127 |
| 68 | Seaman et al. 2018 | Learner background, characteristics | When the results were stratified, by discipline both medicine and nursing students demonstrated significant changes in the total ISVS and subscale scores. Nursing and medical students had similar ISVS scores prior to the placement; however, nursing students demonstrated a larger change in the overall ISVS and the three subscales after the clinical placement. | 3126 |
| 69 | Seaman et al. 2018 | Learner background, characteristics  IPE teaching/learning approaches  Teaching environment: facilitators | Success in terms of acquiring interprofessional skills in placement is likely to be influenced by the understanding and behaviour of the different professionals involved. Our students noted some of the nursing and medical staff did not seem to understand what IPE meant. Establishing professional identity, defined by Oandasan and Reeves (2005) as “a series of attitudes, beliefs and understandings of what that profession means to them, and how they see themselves in a professional role in the future” develops through a process of socialisation. | 3128 |
| 70 | Skolka et al. 2020 | IPE characteristics | Experiences that provide service oriented, IP clinical immersion are effective methods for students to quickly learn how to collaborate and form a functioning team for the benefit of the patients. [24,25,26] | 3 |
| 71 | Skolka et al. 2020 | Learner attitudes, perspectives, behaviors  IPE characteristics  IPE learner development/transformation | There was a shift in attitude post-trip compared to pre-trip. The students began the brigade with unanimously positive, hopeful, idealistic expectations; the students returned from the trip with realistic perspectives. The experience made them realize that IP teamwork was not as easy as anticipated.  Conflicts and misunderstandings arose. They realized that teams require appropriate skills, roles, and respectful interpersonal relationships. | 3-4 |
| 72 | Skolka et al. 2020 | Learner background, characteristics | Although there was no statistical significance between the pre and post-brigade attitudes towards IPE, there was a decline in attitude towards professional identity, teamwork, and collaboration. Patient-centeredness attitudes remained consistently positive. | 2 |
| 73 | Smith et al. 2020 | IPE learner development/transformation | Participants in both groups noted minimal interaction with the other profession prior to the workshop. By understanding one another’s backgrounds and interacting in a safe learning environment, participants gained appreciation for each other’s skills and contributions. | 3083 |
| 74 | Smith et al. 2020 | IPE learner development/transformation | Because of implicit professional hierarchy and limited prior interactions, DMS participants often felt intimidated by physicians and hesitant to share their insights. The opportunity to teach and be recognized for their expertise resulted in more confidence in their teaching and communication skills. | 3084 |
| 75 | Smith et al. 2020 | IPE learner development/transformation | As suggested by the contact theory, bringing IMR and DMS students together within a supportive environment resulted in enhanced respect for one another and increased willingness to engage in future collaborative care. | 3084 |
| 76 | Snyman and Donald 2019 | IPE learner development/transformation | Following the rural rotation, students’ responses regarding their experience of interprofessional holistic patient care varied. Some students reported having had a rich learning experience, while others were unaffected. Those who showed evidence of developmental learning highlighted as key themes (a) an increased knowledge about their own profession’s role, (b) a deeper knowledge about the role of other professions and (c) the importance and value of IPCP. | 331 |
| 77 | Snyman and Donald 2019 | IPE learner development/transformation | Students suggested that being in relationship with people from other health professions outside of professional context – be it family members, friendships, romantic relationships or shared living spaces – had a valuable influence on their increased knowledge and positive perception of other health professionals. (...) Students also commented on the value of getting to know health professionals personally and how that enhanced their interprofessional holistic care. | 331 |
| 78 | Snyman and Donald 2019 | IPE learner development/transformation | Shared living space was raised by students as a factor that enhanced the discovery of other professions’ roles. | 333 |
| 79 | Snyman and Donald 2019 | IPE learner development/transformation | Attending to social learning theory by arranging shared accommodation for students on a distributive training platform and modeling interprofessional collaborative care could also have a positive effect on the outcomes of an interprofessional service-learning initiative. | 334 |
| 80 | Snyman and Donald 2019 | Learner attitudes, perspectives, behaviors | This interprofessional rural service-learning experience had a mixed influence on students’ perception and practice of interprofessional patient care. Some students had positive attitudes prior to the rural placement, some students’ perceptions were positively influenced by the rotation and other students seemed to have come and left with a neutral or even negative attitude toward IPCP. | 332 |
| 81 | Snyman and Donald 2019 | Learner background, characteristics | The findings suggest that students’ attitudes toward IPCP were influenced by their profession, with some professions showing less enthusiasm for IPCP than others. | 332 |
| 82 | Snyman and Donald 2019 | Teaching environment: barriers | There was agreement among students that their experience of the tertiary hospital setting is one of hostility toward interprofessional holistic care. Allied health professions are undermined or neglected to the detriment of patient well-being. Effective communication between different health professionals is lacking and holistic care is generally not well modeled by qualified health professionals. One of the students acknowledged that they were ignorant of the value of interprofessional holistic care until seeing it in action. | 332 |
| 83 | Squires et al. 2020 | Learner background, characteristics | Importantly, none of the physician participants mentioned undergraduate medical education experiences involving IPE. | 195 |
| 84 | Squires et al. 2020 | Learner background, characteristics | It was notable that physician participants in the study were the least likely to have previous work experiences since 90% of them had continued directly from higher education (university level) to medical school. By contrast, the sheer variety of work experiences amongst participants was a testament to the diversity adult learners can bring to the health professions. These work experiences strongly contributed to their perception of IPC and IPE in health care. Participants ranged from 0 to 24 years of previous work experiences and fields included business, the arts, fashion, psychology, K-12 education, military service, retail pharmacy, and non-health care human services, to name a few. The majority of participants had gained their healthcare work experience as a registered nurse in a hospital setting. Home health care nursing, alcohol and drug rehabilitation, health related research assistant, and community-based health care settings were other locations where participants had gained experience that informed their perspectives about IPE and IPC. | 196 |
| 85 | Squires et al. 2020 | Learner background, characteristics | For participants with health care work experience, examples of significant experiences usually came from how care delivery was organized in their facility and if there were the human resources to actually capitalize on IPC. Participants tended to use examples of IPC that drew directly from their own working environments and the professions there. Nurses most frequently cited IPC with physicians and physical therapists, while physicians most frequently discussed IPC with nurse practitioners. | 196 |
| 86 | Squires et al. 2020 | Teaching environment: barriers/facilitators | The role of the organization in shaping attitudes toward IPC and IPE is often deemphasized in the literature. Educators can prepare students to work collaboratively and have those skills, but if the organizational culture does not support the necessary attitudes, behaviors, and competencies for IPC and IPE, most professionals will eventually submit to the organizational norms. This was also evident in how students described where they worked or trained. | 196-197 |
| 87 | Squires et al. 2020 | Teaching environment: barriers/facilitators | We also note that the role of the organization in supporting or negating IPC appears as an important factor in influencing graduate level health professions students’ attitudes and receptivity toward IPC and IPE and may influence program success. (...) It is also important to note that even if students are prepared to be collaborative when they graduate, if the organizational culture does not support IPC and operates under traditional hierarchical, non-team friendly models, then the sustainability of graduates implementing the lessons learned about IPC from these programs is threatened. | 197 |
| 88 | Stephens and Ormandy 2018 | IPE learner development/transformation | Students appreciated the exercises where they learnt about different team roles and how they recognised the roles they themselves take on, as well as the roles that other members of the wider professional team occupy. Students commented on the positive dynamics within the groups, identifying they were better than teams in practice and/or university. | 352 |
| 89 | Stephens and Ormandy 2018 | IPE learner development/transformation | Analysis of the transcripts found that the positive group dynamics had a significant effect on the student’s values, attitudes, and beliefs about each other; that is, they felt they were being treated like a professional. These are intricately linked to the work of Bourdieu (1977) and how social order impacts on the opinions of the students being ready for collaborative learning. Developed from reflecting on the external social world and structures of their professional groups and practices, shaping their sense of their place within an interprofessional team. In the final year, students have learned to navigate the patterns of behaviour expected in the structured social space or field of their profession. | 354 |
| 90 | Stephens and Ormandy 2018 | IPE learner development/transformation | From the focus group comments, it was noted that some students had been affronted by previous practice encounters and saw this as an opportunity to help other professionals internalise new values about them (Epstein, 1977). | 354 |
| 91 | Stephens and Ormandy 2018 | Values towards IPE | The first two stages of development (compliance and iden- tification) are types of conformity and can revert to previously held attitudes and values, as they are both extrinsically moti- vated and require constant reinforcement. However, the third-stage internalisation is when a student embraces the new values and they become part of their belief system (Epstein, 1977). | 355 |
| 92 | Thomson et al. 2020 | IPE learner development/transformation | (...) prior to the session concerns about learning alongside medical students; they felt intimidated and feared there would be a hierarchy, but IPE appeared to be successful in removing these concerns, with nursing students finding the sessions very open and comfortable who also indicated that they found easy to contribute to the session, and they found the group to be very welcoming and respectful, and the session to be very relaxed. The results of this study also suggest that the nursing students became more confident as a result of the teaching; with some indicating that they would be happier to approach a doctor in the future or share information with them. It would appear that IPE resulted in boosting nursing confidence around their medical peers, and decreased concerns about feelings of inferiority/intimidation. Nursing students suggested that the teaching session highlighted the fact that medical students were not so different to nursing. | 5 |
| 93 | Thomson et al. 2020 | IPE learner development/transformation | Medical students found IPE improved their understanding of nursing priorities and thinking, and also illustrated the differences in expertise/skills and roles between the two groups. | 5 |
| 94 | Thomson et al. 2020 | IPE learner development/transformation | Our medical and nursing students showed gains in RIPLs domains, thereby supporting that there was a true benefit from the experience, correlating to the results from other IPE studies.17 The success of this teaching may be also attributed to the nature of geriatrics as a collaborative specialty, and to the educators’ collaborative approach (characteristic for geriatrics problem-solving) in all given cases, contributing both to the students’ positive attitude and to the positive results of the study. | 11 |
| 95 | Thomson et al. 2020 | Learner background, characteristics | IPE had a more positive impact on nursing students, with statistically significant improvements across all aspects of the questionnaire. We speculate that this was probably due to their more extensive clinical experience where they may had already encountered clinical situation needing collaboration between members of the MDT, influencing their perception of their and other professions’ roles, being less ‘observers’, but more ‘workers’ incorporated in their teams. | 11 |
| 96 | Yang et al. 2017 | IPE Characteristics | From the post-course (T2) to end-of-study (T3) period, a significantly greater increase in the total IEPS and ATHCTS scores of the Group 1 (benchmarking) participants than for the Group 2 (regular) participants can be seen. This indicates the additional benefits of IPC benchmarking on the Group 1 participants’ IPC attitude. | 6-8 |
| 97 | Yang et al. 2017 | IPE Characteristics | When trying to improve each health professional’s IPC attitude with limited resources, including the time needed to carry out the training, the number of faculty members needed to run the training and the facilities needed for the training, each newly-trained participant should act as a seed instructor within their team. In other words, successful training of seed instructors can result in profession-wide IPC promotion and attitude remodelling. | 9 |
| 98 | Yang et al. 2017 | IPE learner development/transformation | In open-ended questions at the end of our study, most participants reported that watching the IPE-specific video and discussing it, as well as viewing the uploaded videos on the e-learning platform, markedly encouraged their motivation to improve their IPC attitude. | 6 |
